# Supplementary material for: Dispersal from the Qinghai-Tibet plateau by a high-altitude butterfly is associated with rapid expansion and reorganization of its genome
Source: Nat Commun. 2023 Dec 11;14:8190. doi: 10.1038/s41467-023-44023-2 (PMC10713551; doi:10.1038/s41467-023-44023-2)
Supplement: Supplementary file 1 — Supplementary Information [file 41467_2023_44023_MOESM1_ESM.pdf]

Supplementary Information

Dispersal from the Qinghai-Tibet Plateau by a high-altitude butterfly is associated with rapid expansion and reorganization of its genome

Youjie Zhao, Chengyong Su, Bo He, Ruie Nie, Yunliang Wang, Junye Ma, Jingyu Song, Qun Yang, Jiasheng Hao

This pdf file contains:

Supplementary Figures 1-13

- Supplementary Figure 1. Genome size assessment for 6 *Parnassius* species.
- Supplementary Figure 2. Heatmap of the chromosomal interaction intensity in *P. glacialis* Hi-C assembly.
- Supplementary Figure 3. Chromosomal collinearity between *P. glacialis* and *Pa. bianor*.
- Supplementary Figure 4. TE composition and activity of 3 *Parnassius* butterflies.
- Supplementary Figure 5. Annotation pipeline of complete LTR and Solo-LTRs.
- Supplementary Figure 6. Enriched KEGG pathways for the expanded gene family in *P. glacialis*.
- Supplementary Figure 7. Identification of LTR-mediated RPLP2 pseudogene in *P. glacialis*.
- Supplementary Figure 8. Production of LTR-mediated RPLP2 pseudogene in *P. glacialis*.
- Supplementary Figure 9. Phylogenic tree and LTT plot of RPLP2 genes in 3 *Parnassius* species.
- Supplementary Figure 10. Phylogenetic tree for 9 *P. glacialis* populations at different altitudes.
- Supplementary Figure 11. Cross-validation plot of Admixture analysis.
- Supplementary Figure 12. Maximum likelihood tree of 9 *P. glacialis* populations with migration events.
- Supplementary Figure 13. Recombination rate, TE-SVs and F_{ST} in *P. glacialis*.

Supplementary Tables 1-2

- Supplementary Table 1. Sampling information of 6 *Parnassius* species for genome size assessment.
- Supplementary Table 2. Sampling information and mRNA-Seq statistics of *P. glacialis* populations.

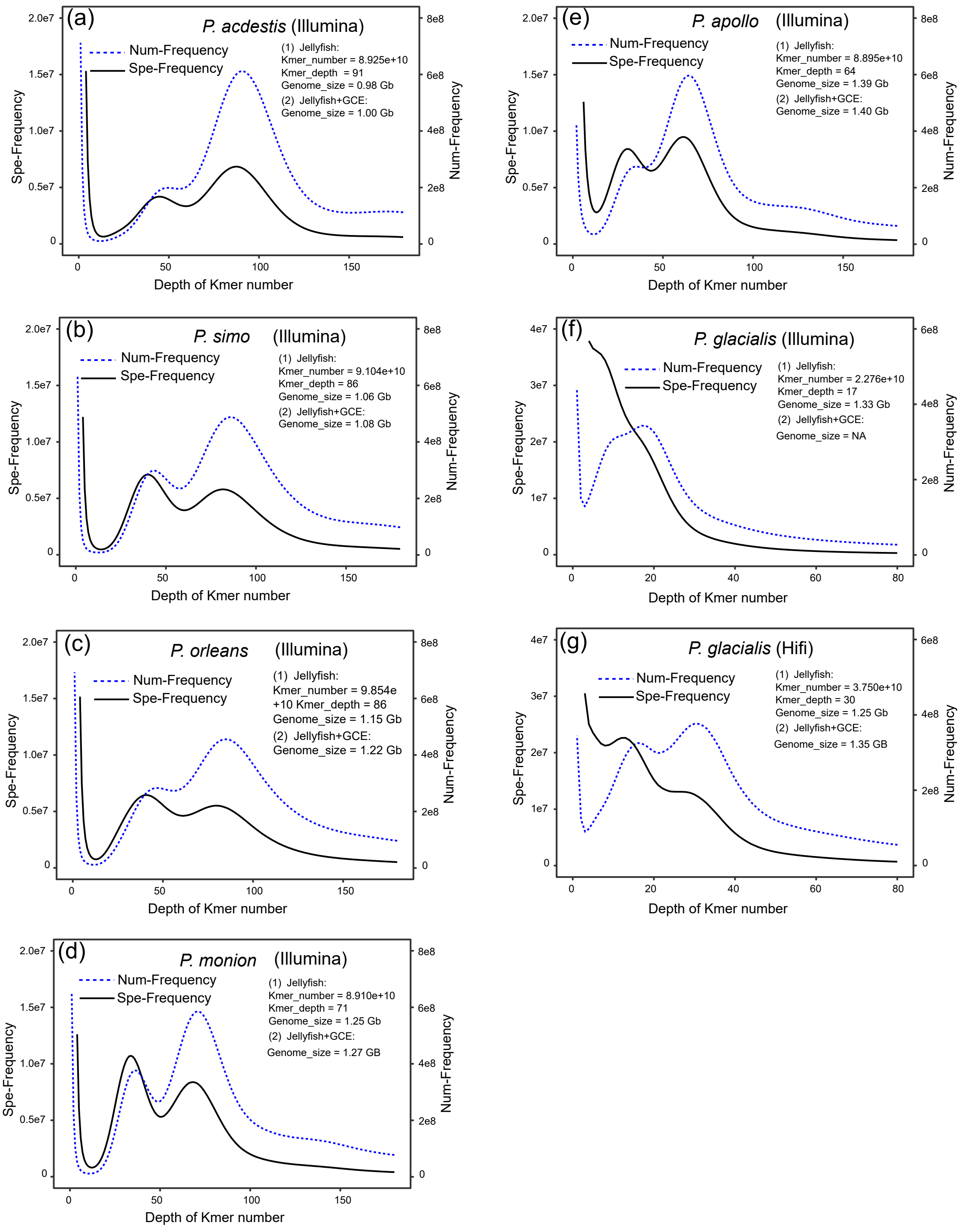

Supplementary Figure 1. Genome size assessment for 6 *Parnassius* species. (a) Assessment for *P. acdestis*. (b) Assessment for *P. simo*. (c) Assessment for *P. orleans*. (d) Assessment for *P. monion*. (e) Assessment for *P. apollo*. (f) Assessment for *P. glacialis* using Illumina sequences. (g) Assessment for *P. glacialis* using PacBio sequences. Black solid line and blue dashed line represent the 17 k-mer Spe-Frequency and Num-Frequency from Jellyfish, respectively. Two methods were used to assess the genome size. (1) Jellyfish: Genome size = Total Kmer_number/Kmer_depth in Num-Frequency. (2) Jellyfish+GCE: Genome size assessment using the Jellyfish and GCE tools. In this study, the genome sizes of 6 *Parnassius* species were mainly referred to the second method (Jellyfish +GCE).

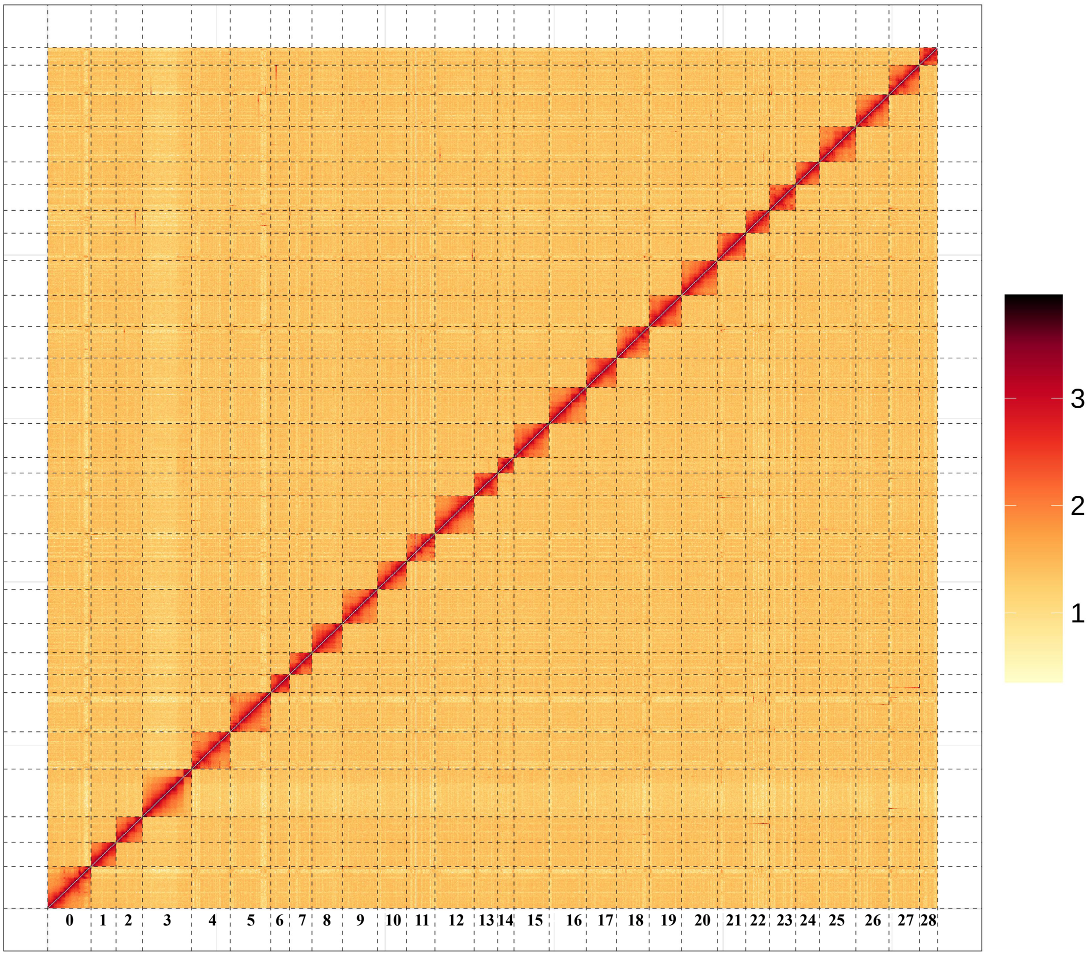

Supplementary Figure 2. Heatmap of the chromosomal interaction intensity in *P. glacialis* Hi-C assembly.

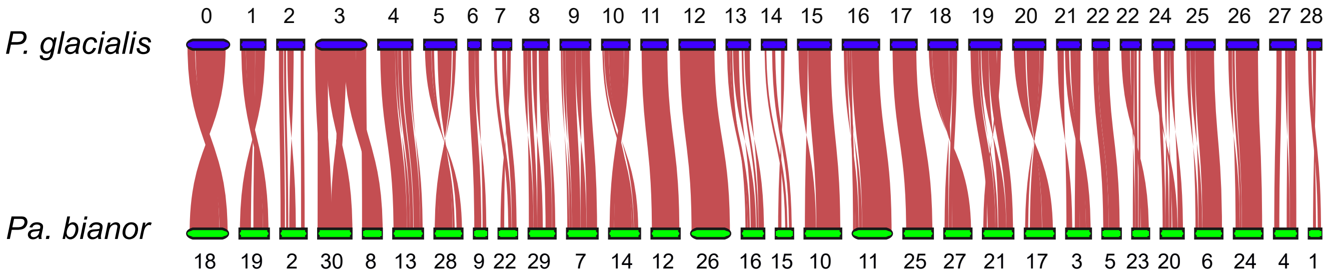

Supplementary Figure 3. Chromosomal collinearity between *P. glacialis* and *Pa. bianor*.

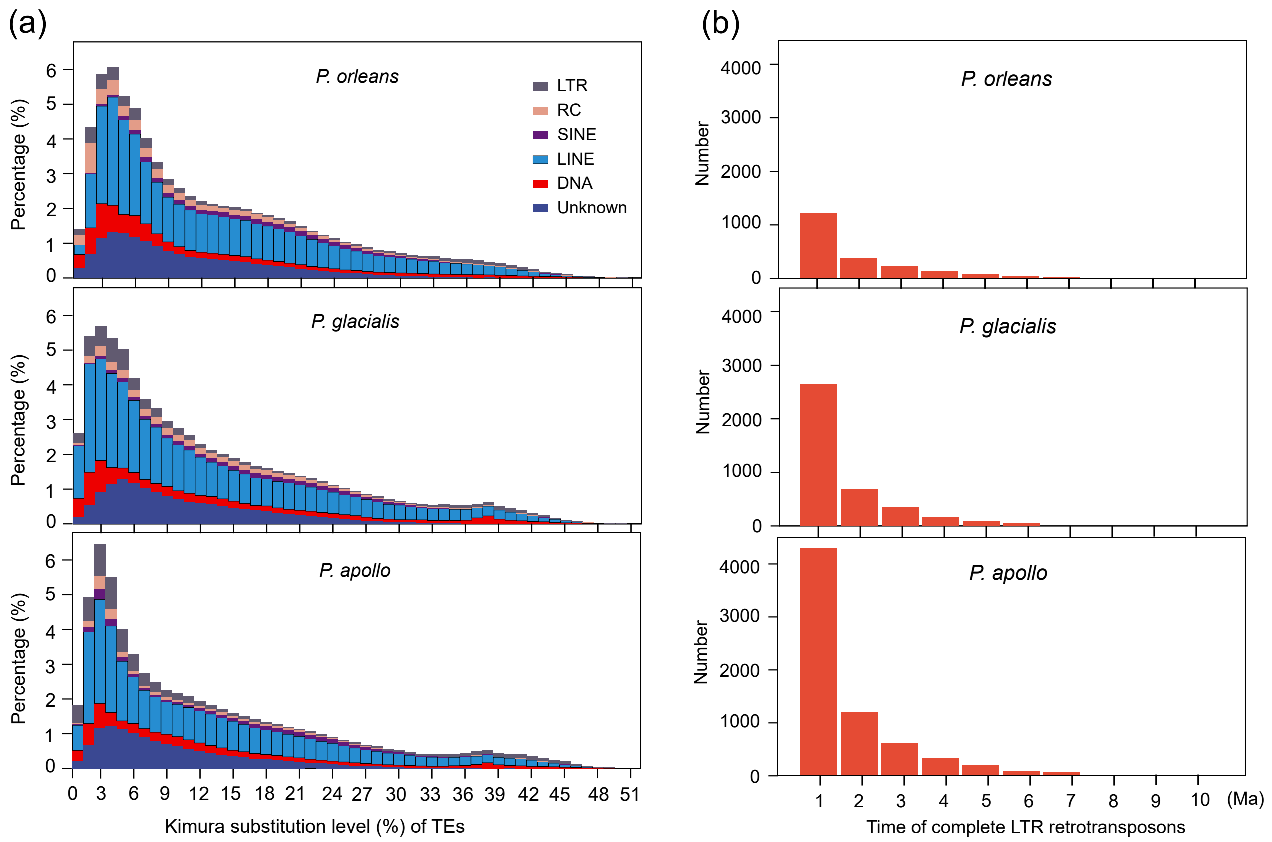

Supplementary Figure 4. TE composition and activity of 3 *Parnassius* butterflies. (a Kimura substitution level (%) of TEs in 3 *Parnassius* species. LTR, RC, SINE, LINE, DNA and unknown stand for different types of TEs. Source data was shown in the source data file (Figure S4a). (b) Age (Ma) of complete LTR retrotransposons in 3 *Parnassius* species. Source data was shown in the source data file (Figure S4b).

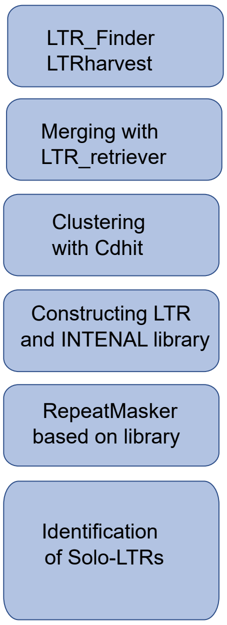

Step1 : LTR_Finder and LTRharvest were used to predict the LTR retrotransposons.

Step2 : LTR_retriever was used to obtain high-quality LTR-RT sequences by merging the results of LTR_Finder and LTRharvest.

Step3 : Cdhit was used to cluster the LTR sequences based on 80-80-80 rule.

Step4 : Clustalw was used to align the 5'LTR and INTERNAL sequences of each family, then a perl script was written to get the consensus sequences of 5'LTR and INTERNAL sequences.

Step5 : RepeatMasker was used to annotate the genome based on the consensus sequences of 5'LTR and INTERNAL sequences.

Step 6 : From the annotation of RepeatMasker, we chosed some LTR-related alignment starting with TG and ending with CA without the INTERNAL sequences of the same family at both flanking 5Kb. Then we checked the upstream and downstream of 5bp region of LTR-related alignment, if 3-5 bp TSD was presented, this LTR-related alignment would be Identified as a Solo-LTR. Several perl scripts were used to employ the process.

Supplementary Figure 5. Annotation pipeline of complete LTR and Solo-LTRs.

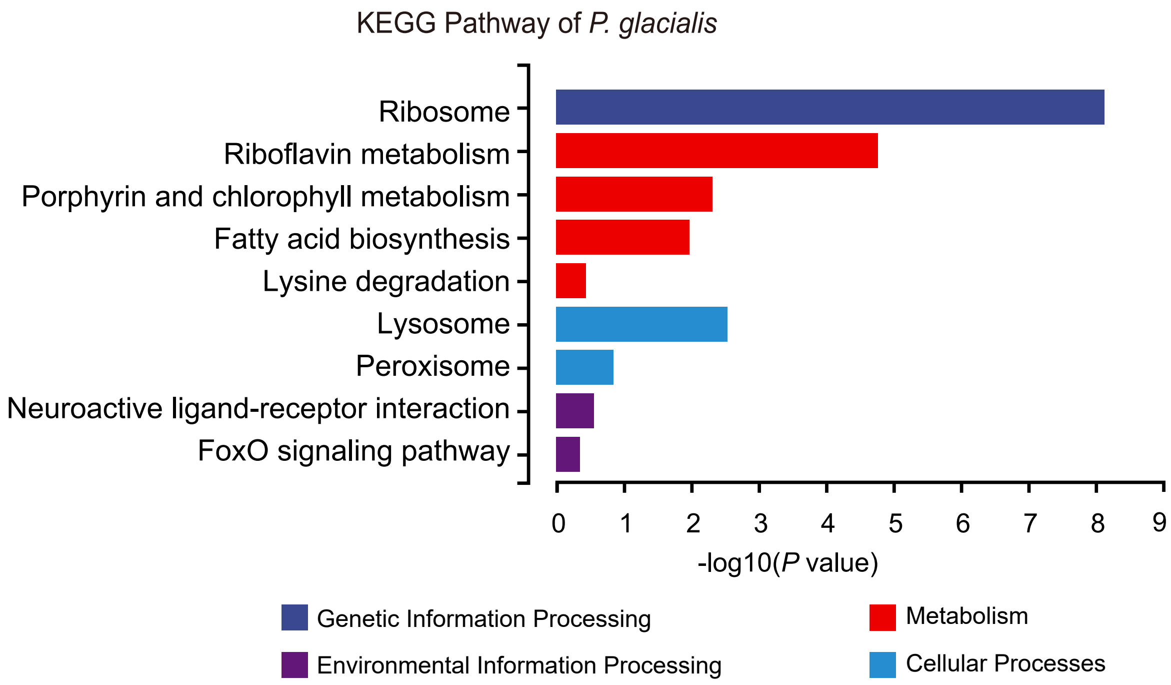

Supplementary Figure 6. Enriched KEGG pathways for the expanded gene family in *P. glacialis*. The exact *P* values were shown in the source data file (Figure S6).

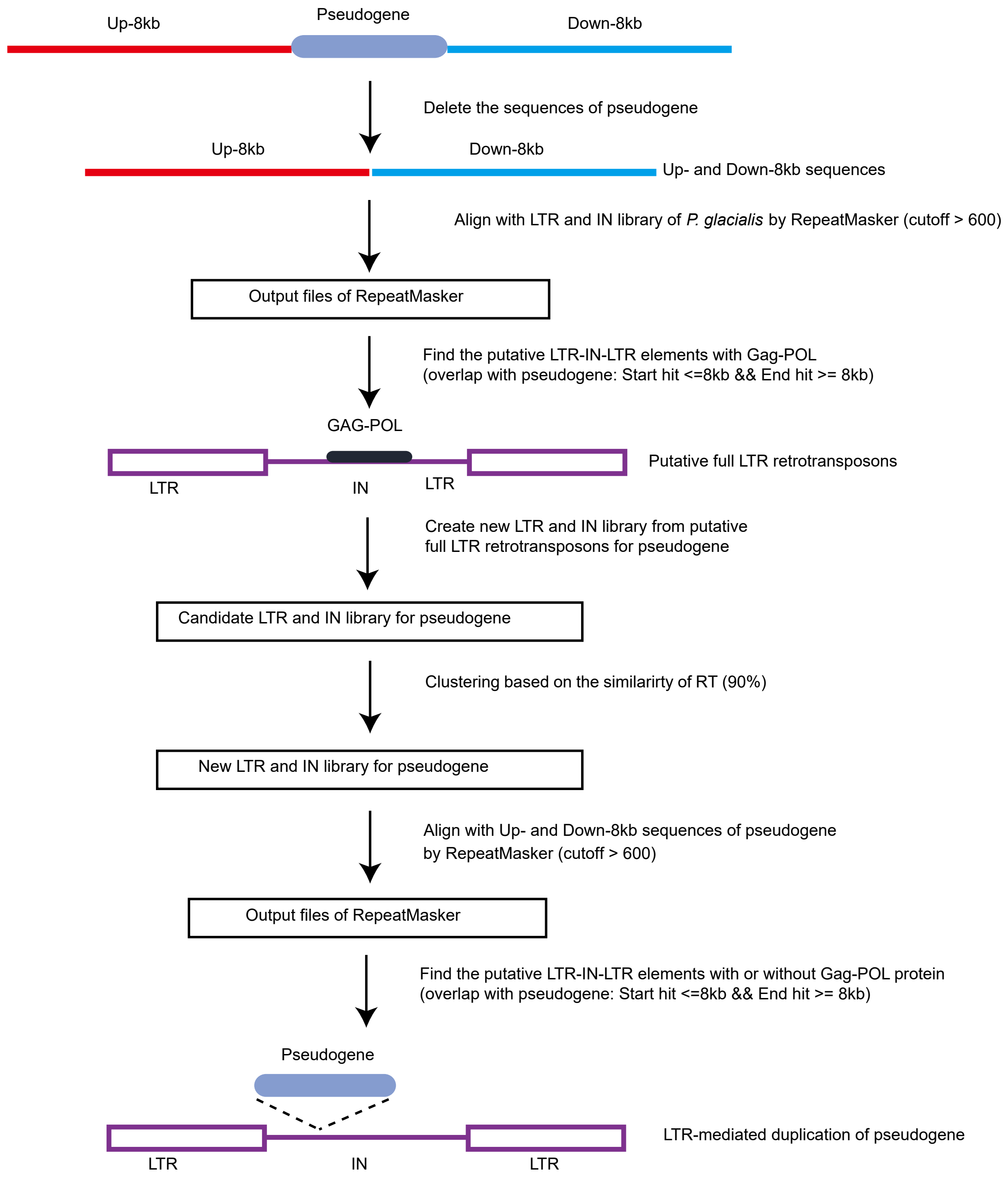

Supplementary Figure 7. Identification of LTR-mediated *RPLP2* pseudogene in *P. glacialis*. Up-8kb: upstream 8kb sequence of pseudogene; Down-8kb: downstream 8kb sequence of pseudogene; LTR: long terminal repeat. IN: INTERNAL region of LTR retrotransposon. GAG-POL: Protein of group-specific antigen and polymerase. RT: reverse transcriptase.

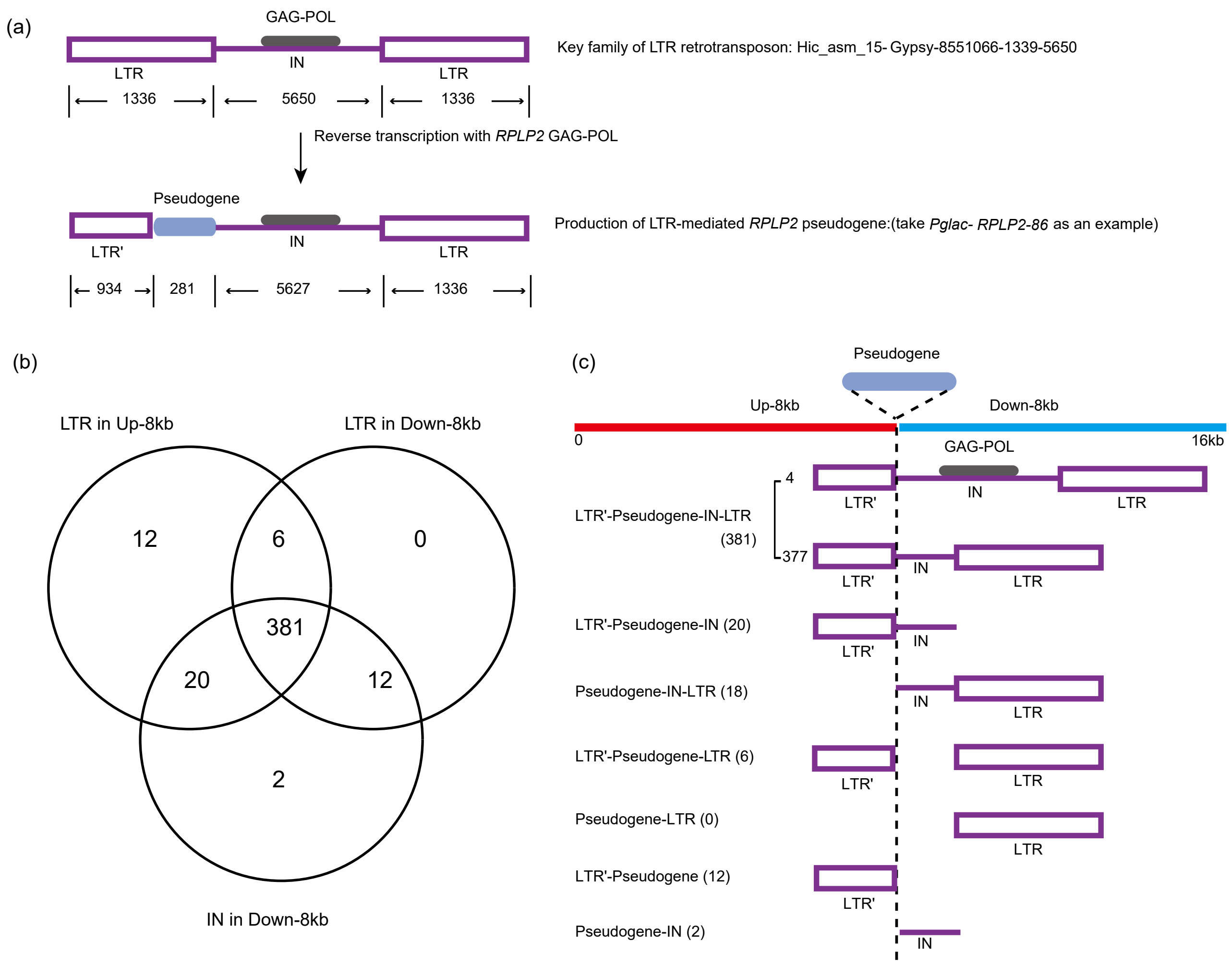

Supplementary Figure 8. Production of LTR-mediated *RPLP2* pseudogenes in *P. glacialis*. (a) Key family of LTR retrotransposon (Hic_asm_15-Gypsy-8551066-1339-5650) responsible for *RPLP2* pseudogene in *P. glacialis*. (b) Alignment position of key family in the Up- and Down-8kb regions of 433 *RPLP2* pseudogenes. For example, 381 *RPLP2* pseudogenes meet the conditions: LTR of the key family exists at Up-8kb region of pseudogene; IN and LTR exist at Down-8kb region (in order of IN-LTR). (c) Different structure of LTR-mediated *RPLP2* pseudogenes. Up-8kb: upstream 8kb sequence of pseudogene; Down-8kb: downstream 8kb sequence of pseudogene; LTR: long terminal repeat; LTR': residual long terminal repeat. IN: INTERNAL region of LTR retrotransposon. GAG-POL: Protein of group-specific antigen and polymerase.

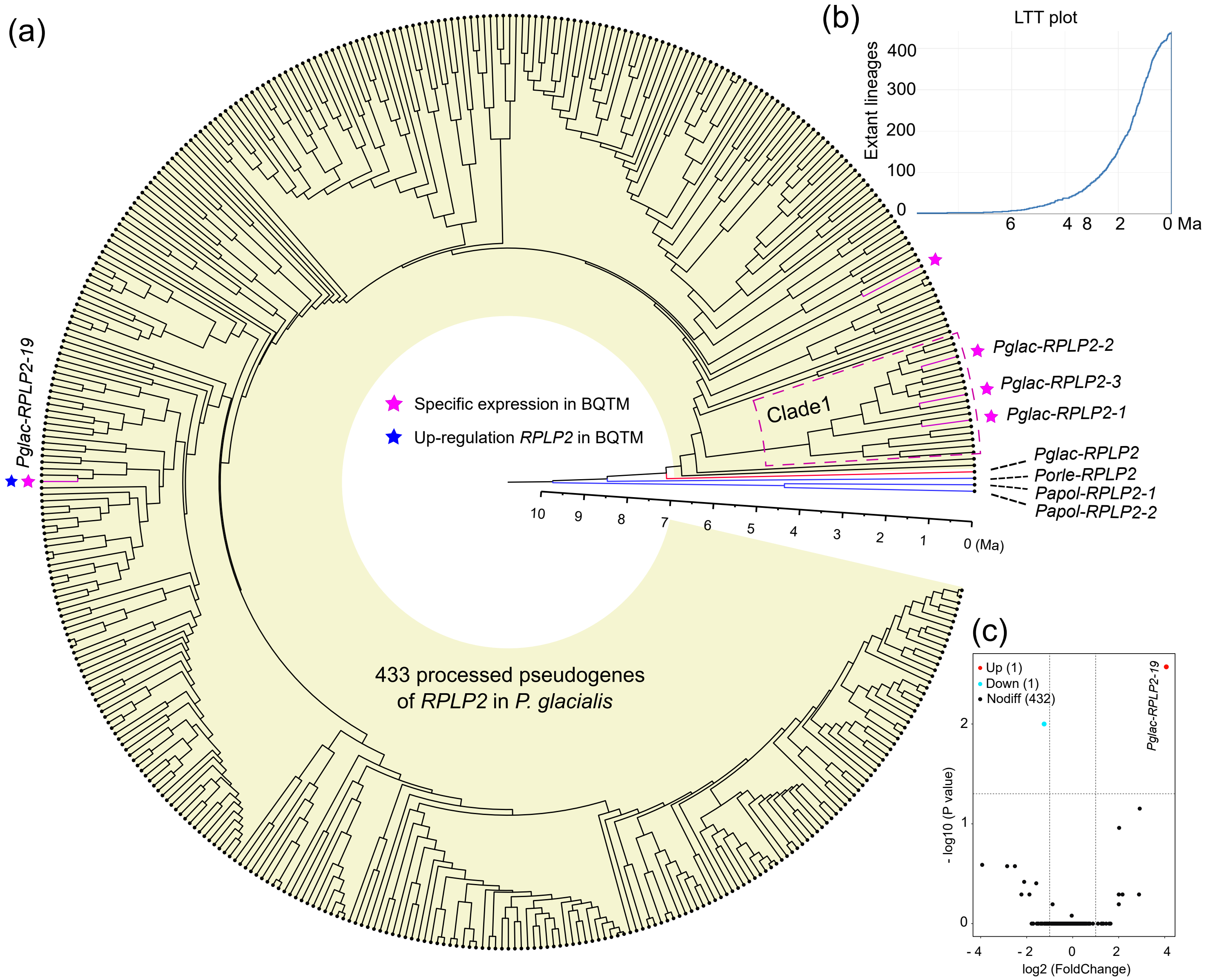

Supplementary Figure 9. Phylogenic tree and LTT plot of *RPLP2* genes in 3 *Parnassius* species. (a) Phylogenic tree of *RPLP2* genes in three *Parnassius* species. The branches with straw yellow color stand for the expansion of 433 processed pseudogenes in *P. glacialis*. *Pglac-RPLP2* is the complete functional *RPLP2* gene in *P. glacialis*. *Porle-RPLP2*, *Papol-RPLP2* and *Papol-RPLP2-1* are the *RPLP2* genes from *P. orleans* and *P. apollo* as the outgroup. Pentagrams represent the specific *RPLP2* pseudogenes with normal expression (Log2(TPM+1)>=1) in the low-altitude population BQTM of *P. glacialis*. Pink pentagram represents the *RPLP2* pseudogenes with specific expression (*Pglac-RPLP2-1*, *Pglac-RPLP2-2*, *Pglac-RPLP2-3* and *Pglac-RPLP2-19*) in the low-altitude population BQTM. Blue pentagram represents the significant Up-regulation *RPLP2* pseudogene (*Pglac-RPLP2-19*) in the low-altitude population BQTM. (b) Lineages-through-time (LTT) plot of *RPLP2* genes in phylogenic tree. (c) Differential expression analysis of *RPLP2* genes between BQTM and BQXL populations for *P. glacialis*. Up (1) represents the up-regulated gene (*Pglac-RPLP2-19*) in BQTM. Down (1) represents the down-regulated gene in BQTM.

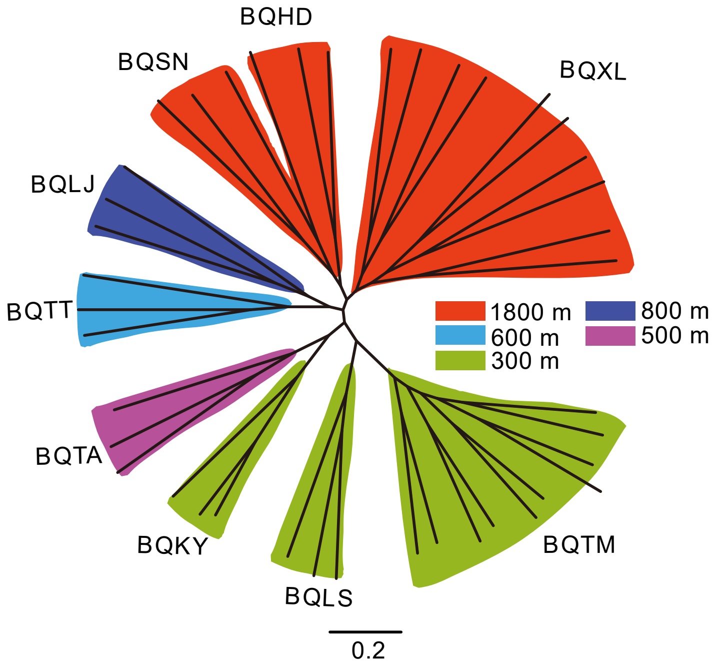

Supplementary Figure 10. Phylogenetic tree for 9 *P. glacialis* populations at different altitudes. Different color strands for different altitude populations.

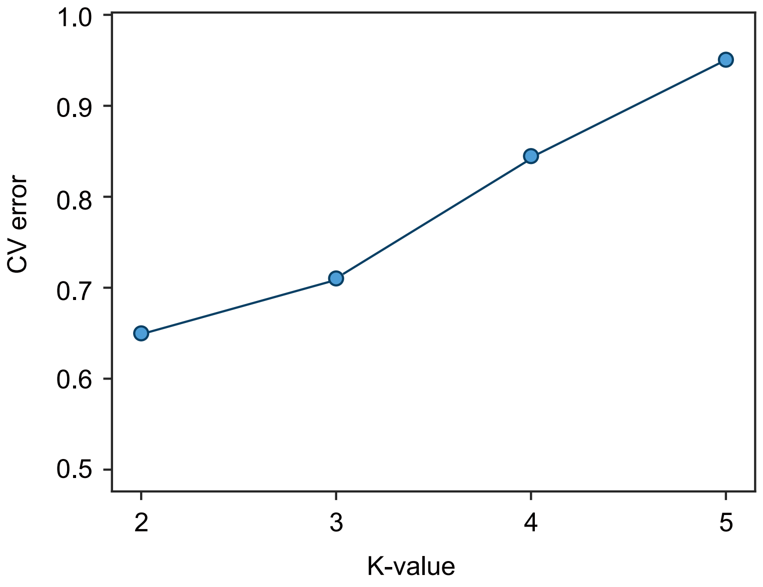

Supplementary Figure 11. Cross-validation plot of Admixture analysis.

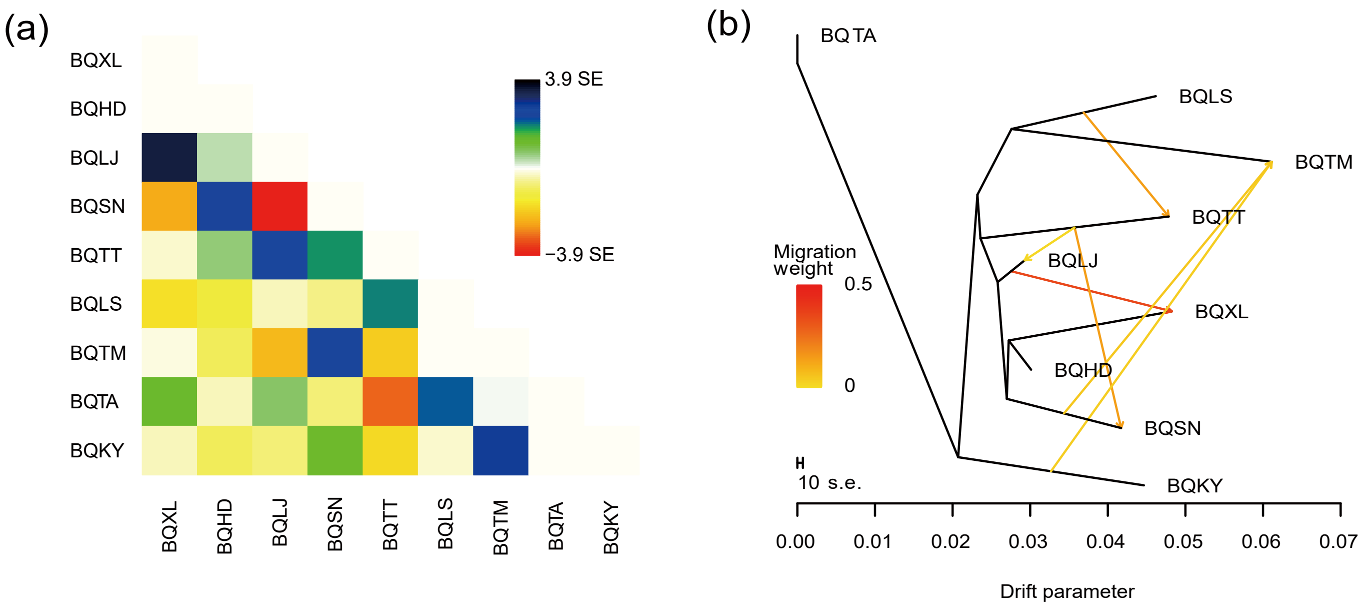

Supplementary Figure 12. Maximum likelihood tree of 9 *P. glacialis* populations with migration events. (a) Residual fit from maximum likelihood tree. (b) Maximum likelihood tree with inferred migration edges.

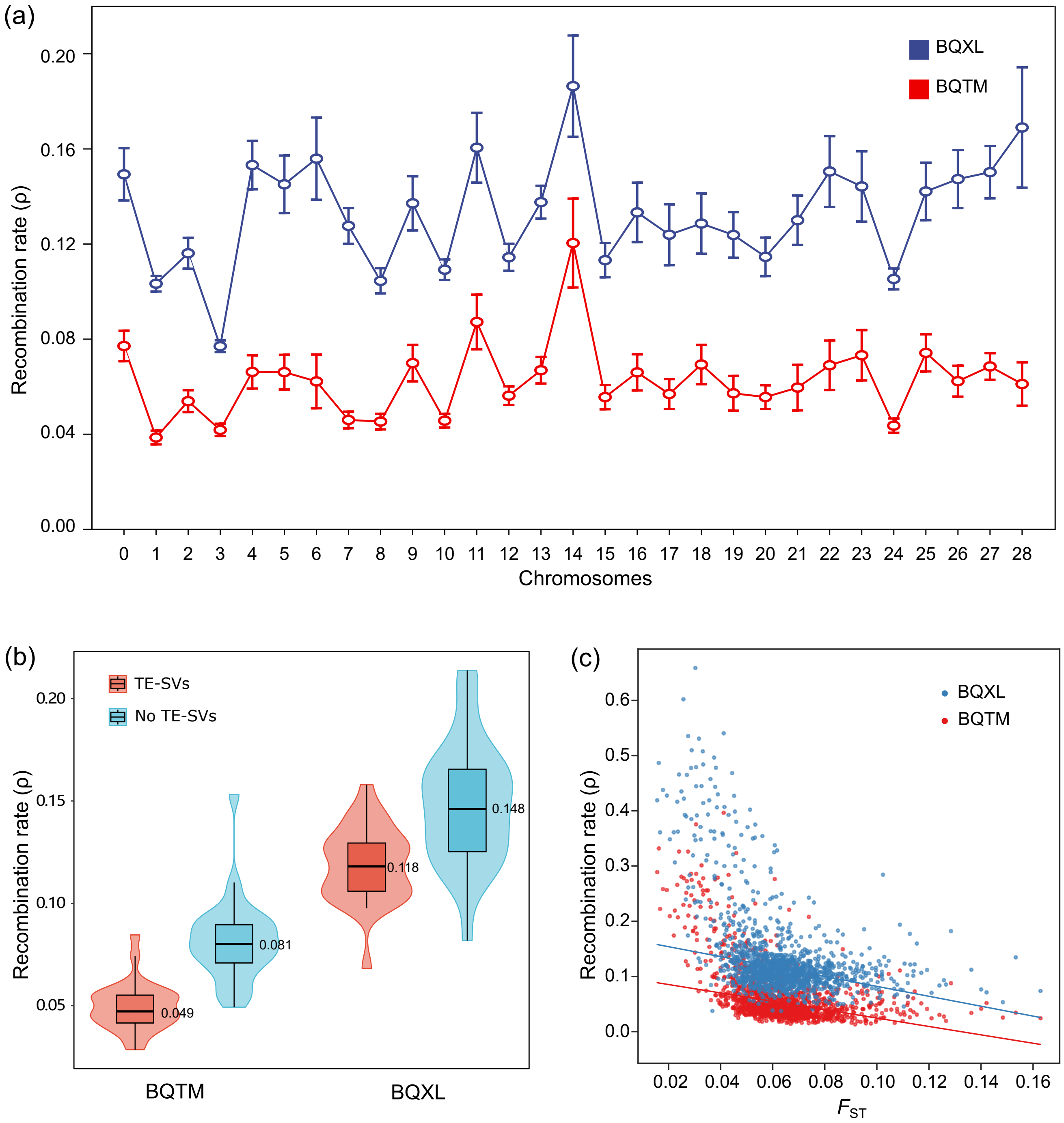

Supplementary Figure 13. Recombination rate, TE-SVs and F_{ST} in *P. glacialis*.
(a) Average recombination rate in BQTM and BQXL populations. The up and down error lines represent the deviation of average recombination rate calculated by all 1-Mb windows for each chromosome. Source data was shown in the source data file (Figure S13a). (b) Comparison of recombination rates with or without TE-SVs in BQTM and BQXL populations (n=29, 29, 29 and 29, left to right, respectively). TE-SVs and No TE-SVs represent the 10-kb genome windows with and without transposon-mediated SVs in 29 chromosomes of *P. glacialis*. For box plots, center lines show the medians; box bounds stand for the 25th and 75th percentiles; whiskers extend 1.5 times the interquartile range from the 25th and 75th percentiles. Source data was shown in the source data file (Figure S13b). (c) Correlation between F_{ST} and recombination rate in *P. glacialis*. Each point represents the average F_{ST} and average recombination rate per 1-Mb genome windows.

Supplementary Table 1. Sampling information of 6 *Parnassius* species for genome size assessment.

Species	Collecting date	Locality	Latitude	Longitude	Altitude (m)	Sequencer	Library layout	Read length (bp)	Clean data (Gb)	Sources
***Parnassius acdestis* Grum-Grshimailo, 1891**	2015/7/15	Bayankalashan, Maduo, Qinghai	97.662	34.114	4889	Illumina HiSeq	Paired-end	150	99.92	SRR20204025
***Parnassius simo* Gray, 1853**	2020/7/14	Elashan, Xinghai, Qinghai	99.512	35.496	4465	Illumina HiSeq	Paired-end	150	101.92	SRR20204008
***Parnassius nomion* Fischer de Waldheim, 1823**	2020/7/17	Kajiaman, Hezuo, Gansu	102.944	35.056	2800	Illumina HiSeq	Paired-end	150	99.75	SRR20204087
***Parnassius orleans* Oberthür, 1890**	2017/7/7	Demulashan, Chayu, Xizang	97.041	29.326	5141	Illumina HiSeq	Paired-end	150	110.30	SRR20204080
***Parnassius apollo* Linnaeus, 1758**	2020/7/2	Hemu, Buerjin, Xinjiang	87.431	48.571	2200	Illumina HiSeq	Paired-end	150	99.58	SRR25606206
***Parnassius glacialis* Butler, 1866**	2021/5/3	Laoshan, Nanjing, Jiangsu	118.837	32.072	300	Illumina HiSeq	Paired-end	150	25.48	This study
***Parnassius glacialis* Butler, 1866**	2021/5/3	Laoshan, Nanjing, Jiangsu	118.837	32.072	300	PacBio HiFi	Single	~20000	37.54	This study

Supplementary Table 2. Sampling information and mRNA-Seq statistics of *P. glacialis* populations

Populations	Sample ID	Locality	Latitude	Longitude	Collecting date	Stage	Tissue	Clean Data (Gb)	Sources
BQXL	BQXL1	Xiaolongshan, Maiji, Gansu	105.68	34.85	2020/6/2	adult	thorax	10.15	SAMN32489569
BQXL	BQXL2	Xiaolongshan, Maiji, Gansu	105.68	34.85	2020/6/2	adult	thorax	8.78	SAMN32489570
BQXL	BQXL3	Xiaolongshan, Maiji, Gansu	105.68	34.85	2020/6/2	adult	thorax	9.39	SAMN32489571
BQTM	BQTM1	Tianmushan, Hangzhou, Zhejiang	119.45	30.34	2020/4/24	adult	thorax	10.01	SAMN32489590
BQTM	BQTM2	Tianmushan, Hangzhou, Zhejiang	119.45	30.34	2020/4/24	adult	thorax	9.64	SAMN32489591
BQTM	BQTM3	Tianmushan, Hangzhou, Zhejiang	119.45	30.34	2020/4/24	adult	thorax	9.42	SAMN32489592
