# Supplementary material for: Dispersal from the Qinghai-Tibet plateau by a high-altitude butterfly is associated with rapid expansion and reorganization of its genome
Source: Nat Commun. 2023 Dec 11;14:8190. doi: 10.1038/s41467-023-44023-2 (PMC10713551; doi:10.1038/s41467-023-44023-2)
Supplement: Supplementary file 3 — Description of Additional Supplementary Data Files [file 41467_2023_44023_MOESM3_ESM.pdf]

Description of Additional Supplementary Files

Supplementary Data 1

Description: TE content of 3 *Parnassius* species.

Supplementary Data 2

Description: Annotation of complete LTR retrotransposons in *Parnassius apollo*.

Supplementary Data 3

Description: Annotation of Solo-LTRs in *Parnassius apollo*.

Supplementary Data 4

Description: Annotation of complete LTR retrotransposons in *Parnassius glacialis*.

Supplementary Data 5

Description: Annotation of Solo-LTRs in *Parnassius glacialis*.

Supplementary Data 6

Description: Annotation of complete LTR retrotransposons in *Parnassius orleans*.

Supplementary Data 7

Description: Annotation of Solo-LTRs in *Parnassius orleans*.

Supplementary Data 8

Description: Information of one functional RPLP2 gene and 433 processed pseudogenes in *Parnassius glacialis*.

Supplementary Data 9

Description: Sequences of RPLP gene family in 9 Lepidoptera species.

Supplementary Data 10

Description: Sequence alignment of 433 processed pseudogenes and complete *RPLP2* genes in 9 Lepidopteran species.

Supplementary Data 11

Description: RepeatMasker of up-8kb and down-8kb for each *RPLP2* pseudogenes.

Supplementary Data 12

Description: Predicted functional sequences of 4 *RPLP2* genes with start codon.

Supplementary Data 13

Description: Sampling information of 9 populations in *Parnassius glacialis*.

Supplementary Data 14

Description: The values of *Pi*, F_{ST} XPEHH and Tajima's D for BQTM and BQXL populations.

Supplementary Data 15

Description: KEGG enrichment of 454 genes with selective signatures in the low-altitude population BQTM.

Supplementary Data 16

Description: Sequences of two Gypsy LTR retrotransposons associated with *RPLP2* pseudogenes.

Supplementary Data 17

Description: Alignment of RT domains from two major gypsy LTR retrotransposons associated with *RPLP2* pseudogenes.
