# Supplementary material for: Dispersal from the Qinghai-Tibet plateau by a high-altitude butterfly is associated with rapid expansion and reorganization of its genome
Source: Nat Commun. 2023 Dec 11;14:8190. doi: 10.1038/s41467-023-44023-2 (PMC10713551; doi:10.1038/s41467-023-44023-2)

```
SequenceID          * strand for the complete RPLP2 genes in 9 Lepidoptera species. Others are processed pseudogenes.

* Bmori-RPLP2       MRYVAAYLLAVLGGKTTPAAADVEKILSSVGIEADAEKLKKVITELNGKDVEQLIAAGREKLSSMPVGGGAP-AAAAAA---APAA----------AAAEEKKEDKKEEKKEDSESDD-DMGFGLFD   127
* Mhype-RPLP2       MRYVAAYLLAVLGGKPSPAAADLEKILSSVGIEADADKLKRVIDELKGKSVEDLMVQGREKLSSMPAGGAAP--AAAAA---APAA----------AAAEEKK-EEKETKKEDSESDDEDMGFGLFD   127
* Dplex-RPLP2       MRYVAAYLLAVLGGKASPAASDLEKILSSVGIEADSERLKKVIDELSGKTVEDLIAQGREKLSAMPAGGAAP--AAAAA---AAPA---------AAAAEEKK-EEKEAKKEESESEDEDMGFGLFD   127
* Prapa-RPLP2       MRYVAAYLLAVLGGKASPAAADIEKILSSVGIEADSEKLKNVISKLEGKTVDDLIAEGREKLSSMPAGGAAP--AAAAA---AAPA----------AAAEDKK-EEKEAKKEESESEDEDMGFGLFD   127
* Pxuth-RPLP2       MRYVAAYLLAVLGGKASPAAADVEKILSSVGIEADSEKLKKVISELNGKSVDELVAQGREKLSSMPVGGGVA--VAAGA--PAAAA----------PAAEEKK-EEKEAKKEESESDDEDMGFGLFD   127
* Pbian-RPLP2       MRYVAAYLLAVLGGKASPAAADVEKILSSVGIEADSEKLKKVISELNGKSVDELVAQGREKLSSMPVGGGVA--VAAGA--PAAAA----------PAAEEKK-EEKEAKKEESESDDEDMGFGLFD   127
* Papol-RPLP2       MRYVAAYLLAVLGGKASPAAADVEKILSSVGIEADSEKLKKVISELNGKSVEELIAQGREKLSSMPVGGAAP--VAAVA---VAAA----------PAAEEKK-EEKEAKKEESESDDEDMGFGLFD   127
  Papol-RPLP2-1     ------MMNTVLGGKASPAAADVERILSSVDIEADSEKLKKVISELNGKSVEEVIAQGREKLSSMPVDGASL--VAAVV---TAAA----------PAAEEKK-EDKETKKEESESDD-GMLNGLRS   127
* Porle-RPLP2       MRYVAAYLLAVLGGKASPAAADVEKILSSVGIEADSEKLKKVISELNGKSVEELIAQGREKLSSMPVGGAAP--VAAVA---AAAA----------PAAEEKK-EEKETKKEESESDDEDMGFGLFD   127
* Pglac-RPLP2       MRYVAAYLLAVLGGKASPAAADVEKILSSVGIEADSEKLKKVISELNGKSVEELIAQGREKLSSMPVGGAAP--VAAVA---AAAA----------PAAEEKK-EEKETKKEESESDDEDMGFGLFD   127
  Pglac-RPLP2-1     -----------------PGAADVEKILSSVGIEADSEKLKKVISELNGKSVEELIAQGREKLSSMPVGGAAP--VAAVA---AAAA----------PAAEEKK-EEKETKKEESESDDEDMVFGLFD   127
  Pglac-RPLP2-2     -----------------PGAADVEKILSSVRIEADSEKLKKVISELNGKSVEELIAQGREKLSSMPVGGAAP--VAAVA---AAAA----------PAAEEKK-EEKETKKEESESDDEDMGFGLFD   127
  Pglac-RPLP2-3     -----------------PGAADVEKILRSVGIEADSEKLKKVISELNGKSVEELIAQGREKLSSMPVGGAAP--VAAVA---AAAA----------PAAEEKK-EEKETKKEESESDDEDMGFGLFD   127
  Pglac-RPLP2-177   --------------------ADVEKILSSVGIEANFEKLKKVISELNGKS-EELIAQGREKLSTMPVGGAAP--VAAVA---AAAA----------PAA-EKK-EEXESKKEESESDDEVMGFGLFD   127
  Pglac-RPLP2-200   --------------------ADVEKILSSVGIEAXSEILKKVISELNCKS-EELIAQGREKLSTMPVGGAAP--VAAVA---AAAA----------PAAE-KK-EEXESKKEESESDDEVMGFGLFD   127
  Pglac-RPLP2-260   -----------------PGAADVEKILSSVGVEANSEKLKKVISELNGKSVEELIAQGREKLSTIPVGGAAP--VAAVA---AAAA----------PAAEKKK-ERXESKKEESESDDEVMGFGLFD   127
  Pglac-RPLP2-50    ------------------------KILNSVGVEANSEKLKKVISELNGKS-EELIAQGREKLSTMSVGGAAP--VAAVA---AAAA----------PVAEKKK-EEXESKKEESESDDEVMGFGLFD   127
  Pglac-RPLP2-208   ------------------------KILNSVGVEANSEKLKKVISELNGKS-EELIAQGREKLSTMPVGGAAP--VAAVA---AAAA----------PAA--KK-EEXESKKEESESDDEVMGFGLFD   127
  Pglac-RPLP2-423   --------------------ADVEKILSSVGIEAXSEILKKVISELNGKS-EELIAQGREKLSTMPVGGAAP--VVAVA---AAAA----------PAAEKKK-EEXESKKEESESDDEDMGFGLFD   127
  Pglac-RPLP2-109   -----------------PGAADVEEILSSVGIEAXSEILKKVISELNGKS-EELIARGREKLSTMPVGGSAP--VAAVA---AAAA----------PAAEEKK-EEKETKKEESXSDDEDMGFGLFD   127
  Pglac-RPLP2-193   ------------------------KILNSVGVEVNSEKLKKVISELNGKS-EELIAQGREKLSTMPVGGAAP--VAAVA---AAAA----------PAAE-KK-EEXESKKEESESDDEVMGFGLFD   127
  Pglac-RPLP2-249   -----------------PGAADVEKILSSVGIEVDSEKLKKVISELNGKS-EELIAQGREKLSTMPVGGAAP--VAVVA---AAAA----------PAAEEKK-EEKETXKEES--DDEDMGFGLFD   127
  Pglac-RPLP2-18    ------------------------KILNSVGVEANSEKLKKVISELNGKS-EELIAQGREKLSTMSVGGAAP--VATVA---AAAA----------PVAEKKK-EERVQEGIXSESDDEVMGFGLFD   127
  Pglac-RPLP2-242   -----------------PGAADVEKILSSVGIEADSEKLKKVISELNGKS-KELIAQGREKLSTMPVGGAAS--VAAVA---AAAA----------PAAEEKK-EEKETKKEESESDDEDMGFGLFD   127
  Pglac-RPLP2-346   -----------------PGAADVEKILSSVGIEADSEXLKKVISELYGKSVEELIAQGREKLSTMPVGGAAP--VAAVA---AAAA----------PAAEKKK-EEXESKKEESESNDEVIGFGLFD   127
  Pglac-RPLP2-29    ------------------------KILNSVGVEANSEKLKKVISELNGKS-EELIAQGREKLSTMSVGGAAP--VAAVA---AAAA----------PVAEKKK-EEXESKKEESESDDEVMGFGLFD   127
  Pglac-RPLP2-406   ------------------GAADVEKILSSVGIEADSEKLKKVISDFYGKSVEELIAQGCEKLSTMPVGGAVP--VAAVA---VAAA----------PAAEEKK-EEXESKKEESESDDEVMGFGLFD   127
  Pglac-RPLP2-369   --------LAVLGGKASQGAADVEKILSSVGIEAXSEILKKFISELNGKS-EELIAQGREKLSTMPVGGVAP--VAAVA---AAAA----------PAAE-KK-EEXESKKEESESDDEVMGFGLFD   127
  Pglac-RPLP2-157   -----------------PGAADVEKILSSVGVEANSEKLKKVISELNGKSVEELIAQGREKLSTMPVGGAAP--VAAVA---AAAA----------PAAEE-K-EEKETKKEESXSDDEDMGFGLFD   127
  Pglac-RPLP2-224   ------------------------KILNSVGVEANSEKLKKVISELNGKS-EELIAQGREKLSTMPVGGAAP--VAAVA---AAAA----------SPLRRRK---RESKKEESESDDEVMGFGLFD   127
  Pglac-RPLP2-94    -----------------PGAADVEKILSSMGIEAEAEKLKKVISELNCMSVGELIAQGREKLLSMPVGGAAP--VAAVA---AAAA----------PAAEEKK-EEKETKKEESESDDEDIGFGLFD   127
  Pglac-RPLP2-317   -----------------PGAADVEKILSSVRFEANFEKLKKVISELNGKSVEELIAQGREKLSTMPVGEAAP--LAAVA---AAAA----------PAAEEKK-EEKETKKEKSXSDDAHMGFGLFN   127
  Pglac-RPLP2-51    --------------------ADVENILNSVGVEANSEKLKKVISELNGKS-EELIAQGREKLSTMPVGEAAP--VAAVA---AAAA----------PAAE-KK-EXRESKKEESESDDEVMGFGLFD   127
  Pglac-RPLP2-199   ------------------------KILNSVGVEANSEKLKKVISELNGKS-EELIAQGREKLSTMPVGEAAP--VAAVA---AAAA----------PAAE-KK-EXRESKKEESESDDEVMGFGLFD   127
  Pglac-RPLP2-275   -----------------PGAADVEKILSSVGIEADSEKLKKVISELNGKS-EELIAQGREKLSTMAVGGAAL--VAAAA---AAAA----------PAAEEKK-EEKETXKEES--DDEDMVFGLFD   127
  Pglac-RPLP2-57    -----------------PGAADVEKILSSVGIEAXSEILKKVISQLNGKS-EELIAQGREKLSTMPVGGAAP--VAAVA---AAAA----------PAAK-KK-EEXESKKEESESDDEVMGFGLFD   127
  Pglac-RPLP2-409   ------------------------KILNSVGVEANSEKLKNVISELNGKS-EELIAQGREKLSTMPVGGAAP--VAAVA---AAAA----------PAGE-KK-EESXSKKEESESEDEVMGFGLFD   127
  Pglac-RPLP2-220   ------------------------RILQARRILQASEKLRKIISELNGKS-EELIAQGREKLSTMPVGGAAP--VAAVA---AATA----------PASEKK--EEKETKKEESXSDYEDMGFGLFD   127
  Pglac-RPLP2-288   ------------------------KILNSVGVEANSEKLKKVISELNGKS-EXLIARGREKLSTMPVGGAAP--VAAVA---AAAA----------PVAEKKK-EEXESEKEESESDDEVMGFSLFD   127
  Pglac-RPLP2-9     -----------------PGAADVEKILSSVGIEADSEKLKKVISELNGKS-EELIAQGREKLSTMPVGGAAP--VAAVA---AAAA----------PAAEEKK-EEKETKKEESESDDEDMGFGLFD   127
  Pglac-RPLP2-147   ------------------------KILNSVGVEANSEKLKKVISELNGKS-EELIAQGREKLSTMPVGGAAP--VAAVA---AAAA----------PAAE-KK-EEXESKKEESESDDEVMGFGLFD   127
  Pglac-RPLP2-172   ------------------------KILNSVGVEANSEKLKKVISKLNGKN-EELIAQGREKLSTMPVGGAAP--GPRSP---LLQR------------RQLRR-RKRESKKEESESDDEVMGFGLFD   127
  Pglac-RPLP2-86    ------------------------KILNSVGVEANSEKLKKVISELNGKS-EELIAQGREKLSTMPVGGAAP--VAAVA---AAAA----------PAAE-KK-EEXESKKEESESDDEVMGFGLFD   127
  Pglac-RPLP2-65    ------------------------KILNSVGVEANSEKLKKVISELNGKS-EELIAQGREKLSTMPVGGAAP--VAAVA---AAAA----------PVAEKKK-EEXESKKEESESDDEVMGFGLFD   127
  Pglac-RPLP2-416   -----------------PGAADVEKILRSVGIEADSEKLKKVISELNGKS-EELIAQGREKLSTMPVGGAVP--VAAVA----AAA----------PAAEKK--EEXESKKEESESDDEVMGFGLFD   127
  Pglac-RPLP2-372   -----------------PGAADVEKILSSVGIDANSEKLKKVISELNGKNVEELIAQGREKLSTMPVGGAAP--VAAVA---AAAA----------SAAEEKK-EEKGTXKEES--DDEDMGFGLFD   127
  Pglac-RPLP2-78    ------------------------KILNSVGVEANSEKLKKVISELNGKS-EELIAQGREKLSTMSVGGAAP--VAAVA---AAAA----------PVAEKKK-EEXESKKEESESDDEVMGFGLFD   127
  Pglac-RPLP2-40    -----------------PGAADVEKILSSVGIEADSEKLKKVISELNGKSVEELIAQGREKLSTMPVGGAAP--VAAVA---AAAA----------PAAKEKK-EEKETKKEESVSDNEDMGFGLFD   127
  Pglac-RPLP2-124   ------------------------KILNSVGVEANSEKLKKVISELNGKS-EELIAQGREKLSTMSVGGAAP--VAAVA---AAAA----------PVAEKKK-EEXESKKEESESVDEVMGFGLFD   127
  Pglac-RPLP2-261   -----------------PGAADVEKILSSVGIEAXSEILKEVISELNGKS-EELIAQGREKLSTMPVGGAAP--VSAVA---AAAA----------PAAEKKK-EEXESKKEESESDDEDMGFGLFD   127
  Pglac-RPLP2-83    ------------------------KILNSVGVEANSEKLKKVISELNGKS-EELIAQGREKLSTMSVGGAAP--VAAVA---AAAA----------PAAE-KK-EEXESKKEESESDDEVMGFGLFD   127
  Pglac-RPLP2-228   -----------------PGAADVEKILSSMGIEADSEKLKKVISELNAKSVEELIAQGREKLSSMPVGGAAP--VAAVA---ATAA----------PAAEEKK-EEKETKKEESESDDEDMGFGLFD   127
  Pglac-RPLP2-75    ------------------------KILNSVGVEANSEKLKKVISELNGKS-EELIAQGRQKLTTMSVGGAAP--VAAVA---AAAA----------PVAEKKK-EEXESKKEESESDDEVMGFGLFD   127
  Pglac-RPLP2-258   ------------------------KILNSVGVEANSEKLKKVISELNGKS-EELIAQGREKLSTMPVGGAAP--VAAVA---VAAA----------PAAE--K-KEXESKKEESESDDEVMGFGLFD   127
  Pglac-RPLP2-184   -----------------PGAADVEKILSSVGIEADSEKLKKVISELNGKS-EELIAQGREKLSTMPVGGAAP--VXAVA---AAVA----------PAAEEKK-EEKETKKEESXSDDEDMGFGLFD   127
  Pglac-RPLP2-425   ------------------------KILNSVGVEANSEKLKKVISELNGKS-EELIAQGREKLSTMPVGEAAP--VAAVA---AAAA----------PAAE-KK-EXRESKKEESESDDEVMGFGLFD   127
  Pglac-RPLP2-411   -----------------PGAADVEKILSSVCIEANSEKLKKVISELNGKNVEELIAQGREKLSTMLVGEAAP--VAAVA---ATAA----------PAAEEKK-EEXESKKEESESDDEVMGFGLFD   127
  Pglac-RPLP2-374   -----------------PGAADVEKILSSVGIEADSEKLKKVISELNGKS-DELIAQGREKLSTMPVGGAAP--VAAVA---AAAA----------PAAEEKK-EEKETKKEKSXSDDEDMGFVLFD   127
                    1.......10........20........30........40........50........60........70........80........90.......100.......110.......120.......
```

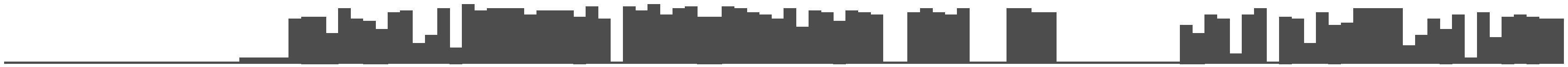

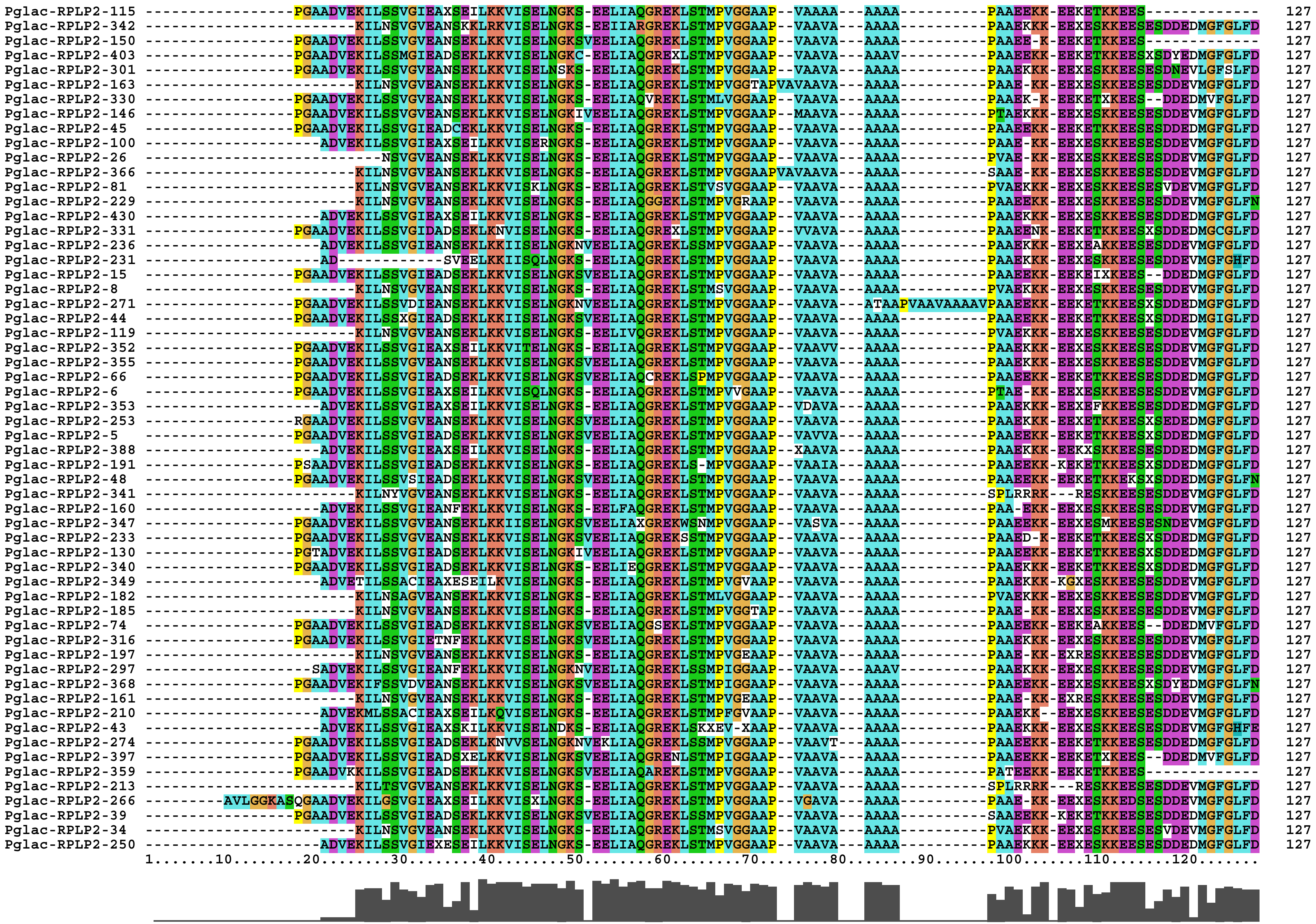

Pglac-RPLP2-115 -----------------PGAADVEKILSSVGIEAXSEILKKVISELNGKS-EELIAQGREKLSTMPVGGAAP--VAAAA---AAAA----------PAAEEKK-EEKETKKEES------------ 127
Pglac-RPLP2-342 ------------------------KILNSVGVEANSKKLRKVISELNGKS-EEIIARGREKLSTMPVGGAAP--VAAVA---AAAA----------PAAEKKK-EEXESKKEESESDDEDMGFGLFD 127
Pglac-RPLP2-150 -----------------PGAADVEKILSSVGVEANSEKLKKVISELNGKSVEELIAQGREKLSTMPVGGAAP--VAAVA---AAAA----------PAAEE-K-EEKETKKEES------------ 127
Pglac-RPLP2-403 -----------------PGAADVEKILSSMGIEADSEKLKKVISELNGKC-EELIAQGREXLSTMPVGGAAP--VAAVA---AAAV----------PAAEEKK-EEKETKKEESXSDYEDMGFGLFD 127
Pglac-RPLP2-301 -----------------PGAADVEKILSSVGVEANSEKLKKVISELNSKS-EELIAQGREKLSTMPVGGAAP--VAAVA---AAAA----------PAAEKKK-EEXESKKEESESDNEVLGFSLFD 127
Pglac-RPLP2-163 ------------------------KILNSVGVEANSEKLKKVISELNGKS-EELIAQGREKLSTMPVGGTAPVAVAAVA---AAAA----------PAAE-KK-EEXESKKEESESDDEVMGFGLFD 127
Pglac-RPLP2-330 -----------------PGAADVEKILSSVGVEANSEKLKKVISELNGKS-EELIAQVREKLSTMLVGGAAP--VAAVA---AAAA----------PAAEK-K-EEKETXKEES--DDEDMVFGLFD 127
Pglac-RPLP2-146 -----------------PGAADVEKILSSVGVEANSEKLKKVISELNGKIVEELIAQGREKLSTMPVGGAAP--MAAVA---AAAA----------PTAEKKK-EEXESKKEESESDDEVMGFGLFD 127
Pglac-RPLP2-45 -----------------PGAADVEKILSSVGIEADCEKLKKVISELNGKS-EELIAQGREKLSTMPVGGAAP--VAAVA---AAAA----------PAAEEKK-EEKETKKEESESDDEDMGFGLFD 127
Pglac-RPLP2-100 --------------------ADVEKILSSVGIEAXSEILKKVISERNGKS-EELIAQGREKLSTMPVGGAAP--VAAVA---AAAA----------PAAE-KK-EEXESKKEESESDDEVMGFGLFD 127
Pglac-RPLP2-26 ---------------------------NSVGVEANSEKLKKVISELNGKS-EELIAQGREKLSTMPVGGAAP--VAAVA---AAAA----------PVAE-KK-EEXESKKEESESDDEVMGFGLFD 127
Pglac-RPLP2-366 ------------------------KILNSVGVEANSEKLKKVISELNGKS-EELIAQGREKLSTMPVGGAAPVAVAAVA---AAAA----------SAAE-KK-EEXESKKEESESDDEVMGFGLFD 127
Pglac-RPLP2-81 ------------------------KILNSVGVEANSEKLKKVISKLNGKS-EELIAQGREKLSTVSVGGAAP--VAAVA---AAAA----------PVAEKKK-EEXESKKEESESVDEVMGFGLFD 127
Pglac-RPLP2-229 ------------------------KILNSVGVEANSEKLKKVISELNGKS-EELIAQGGEKLSTMPVGRAAP--VAAVA---AAAA----------PAAEEKK-EEXESKKEESESDDEVMGFGLFN 127
Pglac-RPLP2-430 --------------------ADVEKILSSVGIEAXSEILKKVISELNGKS-EELIAQGREKLSTMPVGGAAP--VAAVA---AAAA----------PAAEKKK-EEXESKKEESESDDEDMGFGLFD 127
Pglac-RPLP2-331 -----------------PGAADVEKILSSVGIDADSEKLKNVISELNGKS-EELIAQGREXLSTMPVGGAAP--VVAVA---AAAA----------PAAEENK-EEKETKKEESXSDDEDMGCGLFD 127
Pglac-RPLP2-236 --------------------ADVEKILSSVGIEANSEKLKKIISELNGKNVEELIAQGREKLSSMPVGGAAP--VAAVA---AAAA----------PAAEKKK-EEXEAKKEESESDDEVMGFGLFD 127
Pglac-RPLP2-231 --------------------AD-----------SVEELKKIISQLNGKS-EELIAQGREKLSTMPVGGAAP--VAAVA---AAAA----------PAAEKKK-EEXESKKEESESDDEVMGFGHFD 127
Pglac-RPLP2-15 -----------------PGAADVEKILSSVGIEADSEKLKKVISELNGKSVEELIAQGREKLSTMPVGGAAP--VAAVA---AAAA----------PAAEEKK-EEKEIXKEES--DDEDMGFGLFD 127
Pglac-RPLP2-8 ------------------------KILNSVGVEANSEKLKKVISELNGKS-EELIAQGREKLSTMSVGGAAP--VAAVA---AAAA----------PVAEKKK-EEXESKKEESESDDEVMGFGLFD 127
Pglac-RPLP2-271 -----------------PGAADVEKILSSVDIEANSEKLKKVISELNGKNVEELIAQGREKLSTMPVGGAAP--VAAVA---ATAAPVAAVAAAAVPAAEEKK-EEKETKKEESXSDDEDMGFGLFD 127
Pglac-RPLP2-44 -----------------PGAADVEKILSSXGIEADSEKLKKIISELNGKSVEELIAQGREKLSTMPVGGAAP--VAAVA---AAAA----------PAAEEKK-EEKETKKEESXSDDEDMGIGLFD 127
Pglac-RPLP2-119 ------------------------KILNSVGVEANSEKLKKVISELNGKS-EELIVQGREKLSTMPVGGAAP--VAAVA---AAAA----------PVAEKKK-EEXESKKEESESDDEVMGFGLFD 127
Pglac-RPLP2-352 -----------------PGAADVEKILSSVGIEAXSEILKKVITELNGKS-EELIAQGREKLSTMPVGGAAP--VAAVV---AAAA----------PAAEKKK-EEXESKKEESESDDEVMGFGLFD 127
Pglac-RPLP2-355 -----------------PGAADVEKILSSVGVEANSEKLKKVISELNGKSVEELIAQGREKLSTMPVGGAAP--VAAVA---AAAA----------PAAEKKK-EEXESKKEESESDDEVMGFGLFD 127
Pglac-RPLP2-66 -----------------PGAADVEKILSSVGIEADSEKLKKVISELNGKSVEELIAQCREKLSPMPVGGAAP--VAAVA---AAAA----------PAAEEKK-EEKETKKEESESDDEDMGFGLFD 127
Pglac-RPLP2-6 -----------------PGAADVEKILSSVGIEAXSEILKKVISQLNGKS-EELIAQGREKLSTMPVVGAAP--VAAVA---AAAA----------PTAE-KK-EEXESKKEESESDDEVMGFGLFD 127
Pglac-RPLP2-353 --------------------ADVEKILSSVGIEAXSEILKKVISELNGKS-EELIAQGREKLSTMPVGGAAP--VDAVA---AAAA----------PAAEKKK-EEXEFKKEESESDDEDMGFGLFD 127
Pglac-RPLP2-253 -----------------RGAADVEKILSSVGVEANSEKLKKVISELNGKSVEELIAQGREKLSTMPVGGAAP--VAAVA---AAAA----------PAAEKKK-EEXETKKEESXSEDEDMGFGLFD 127
Pglac-RPLP2-5 -----------------PGAADVEKILSSVGIEADSEKLKKVISELNGKSVEELIAQGREKLSTMPVGGAAP--VAVVA---AAAA----------PAAEEKK-EEKETKKEESXSDDEDMGFGLFD 127
Pglac-RPLP2-388 --------------------ADVEKILSSVGIEAXSEILKKVISELNGKS-EELIAQGREKLSTMPVGGAAP--XAAVA---AAAA----------PAAEKKK-EEKXSKKEESESDDEDMGFGLFD 127
Pglac-RPLP2-191 -----------------PSAADVEKILSSVGIEADSEKLKKVISELNGKS-EELIAQGREKLS-MPVGGAAP--VAAIA---AAAA----------PAAEEKK-KEKETKKEESXSDDEDMGFGLFD 127
Pglac-RPLP2-48 -----------------PGAADVEKILSSVSIEADSEKLKKVISELNGKSVEELIAQGREKLSTMPVGGAAP--VAAVA---AAAA----------PAAEEKK-EEKETKKEKSXSDDEDMGFGLFN 127
Pglac-RPLP2-341 ------------------------KILNYVGVEANSEKLKKVISELNGKS-EELIAQGREKLSTMPVGGAAP--VAAVA---AAAA----------SPLRRRK---RESKKEESESDDEVMGFGLFD 127
Pglac-RPLP2-160 --------------------ADVEKILSSVGIEANFEKLKKVISELNGKS-EELFAQGREKLSTMPVGGAAP--VAAVA---AAAA----------PAA-EKK-EEXESKKEESESDDEVMGFGLFD 127
Pglac-RPLP2-347 -----------------PGAADVEKILSSVGVEANSEKLKKIISELNGKSVEELIAXGREKWSNMPVGGAAP--VASVA---AAAA----------PAAEEKK-EEXESMKEESESNDEVMGFGLFD 127
Pglac-RPLP2-233 -----------------PGAADVEKILSSVGVEANSEKLKKVISELNGKSVEELIAQGREKSSTMPVGGAAP--VAAVA---AAAA----------PAAED-K-EEKETKKEESXSDDEDMGFGLFD 127
Pglac-RPLP2-130 -----------------PGTADVEKILSSVGIEADSEKLKKVISELNGKIVEELIAQGREKLSTMPVGGAAP--VAAVA---AAAA----------PAAEEKK-EEKETKKEESXSDDEDMGFGLFD 127
Pglac-RPLP2-340 -----------------PGAADVEKILSSVGIEADSEKLKKVISELNGKS-EELIEQGREKLSTMPVGGAAP--VAAVA---AAAA----------PAAEKKK-EEKETKKEESXSDDEDMGFGLFD 127
Pglac-RPLP2-349 --------------------ADVETILSSACIEAXESEILKVISELNGKS-EELIAQGREKLSTMPVGVAAP--VAAVA---AAAA----------PAAEKKK-KGXESKKEESESDDEVMGFGLFD 127
Pglac-RPLP2-182 ------------------------KILNSAGVEANSEKLKKVISELNGKS-EELIAQGREKLSTMLVGGAAP--VAAVA---AAAA----------PVAEKKK-EEXESKKEESESDDEVMGFGLFD 127
Pglac-RPLP2-185 ------------------------KILNSVGVEANSEKLKKVISELNGKS-EELIAQGREKLSTMPVGGTAP--VAAVA---AAAA----------PAAE-KK-EEXESKKEESESDDEVMGFGLFD 127
Pglac-RPLP2-74 -----------------PGAADVEKILSSVGIEADSEKLKKVISELNGKSVEELIAQGSEKLSTMPVGGAAP--VAAVA---AAAA----------PAAEEKK-EEKEAKKEES--DDEDMVFGLFD 127
Pglac-RPLP2-316 -----------------PGAADVEKILSSVGIETNFEKLKKVISELNGKSVEELIAQGREKLSTMPVGGAAP--VAAVA---AAAA----------PAAEKKK-EEXESKKEESESDDEVMGFGLFD 127
Pglac-RPLP2-197 ------------------------KILNSVGVEANSEKLKKVISELNGKS-EELIAQGREKLSTMPVGEAAP--VAAVA---AAAA----------PAAE-KK-EXRESKKEESESDDEVMGFGLFD 127
Pglac-RPLP2-297 -------------------SADVEKILSSVGIEANFEKLKKVISELNGKNVEELIAQGREKLSSMPIGGAAP--VAAVA---AAAV----------PAAEKKK-EEXESKKEESESDDEVMGFGLFD 127
Pglac-RPLP2-368 -----------------PGAADVEKIFSSVDVEANSEKLKKVISELNGKSVEELIAQGREKLSTMPIGGAAP--VAAVA---AAAA----------PAAEEKK-EEXESKKEESXSDYEDMGFGLFN 127
Pglac-RPLP2-161 ------------------------KILNSVGVEANSEKLKKVISELNGKS-EELIAQGREKLSTMPVGEAAP--VAAVA---AAAA----------PAAE-KK-EXRESKKEESESDDEVMGFGLFD 127
Pglac-RPLP2-210 --------------------ADVEKMLSSACIEAXSEILKQVISELNGKS-EELIAQGREKLSTMPFGVAAP--VAAVA---AAAA----------PAAEKK--EEXESKKEESESDDEVMGFGLFD 127
Pglac-RPLP2-43 --------------------ADVEKILSSVGIEAXSKILKKVISELNDKS-EELIAQGREKLSKXEV-XAAP--VAAVA---AAAA----------PAAEKKK-EEXESKKEESESDDEVMGFGHFE 127
Pglac-RPLP2-274 -----------------PGAADVEKILSSVGIEADSEKLKNVVSELNGKNVEKLIAQGREKLSSMPVGGAAP--VAAVT---AAAA----------PAAEEKK-EEKETKKEESESDDEDMGFGLFD 127
Pglac-RPLP2-397 -----------------PGAADVEKILSSVGIEADSXELKKVISELNGKSVEELIAQGRENLSTMPIGGAAP--VAAVA---AAAA----------PAAEEKK-EEKETXKEES--DDEDMVFGLFD 127
Pglac-RPLP2-359 -----------------PGAADVKKILSSVGIEADSEKLKKVISELNGKSVEELIAQAREKLSTMPVGGAAP--VAAVA---AAAA----------PATEEKK-EEKETKKEES------------ 127
Pglac-RPLP2-213 ------------------------KILTSVGVEANSEKLKKVISELNGKS-EELIAQGREKLSTMPVGGAAP--VAAVA---AAAA----------SPLRRRK---RESKKEESESDDEVMGFGLFD 127
Pglac-RPLP2-266 --------AVLGGKASQGAADVEKILGSVGIEAXSEILKKVISXLNGKS-EELIAQGREKLSTMPVGGAAP--VGAVA---AAAA----------PAAE-KK-EEXESKKEDSESDDEVMGFGLFD 127
Pglac-RPLP2-39 -----------------PGAADVEKILSSVGIEADSEKLKKVISELNGKSVEELIAQGREKLSSMPVGGAAP--VAAVA---AAAA----------SAAEEKK-KEKETKKEESESDDEDMGFGLFD 127
Pglac-RPLP2-34 ------------------------KILNSVGVEANSEKLKKVISELNGKS-EELIAQGREKLSTMSVGGAAP--VAAVA---AAAA----------PVAEKKK-EEXESKKEESESVDEVMGFGLFD 127
Pglac-RPLP2-250 --------------------ADVEKILSSVGIEXESEILKKVISELNGKS-EELIAQGREKLSTMPVGGAAP--VAAVA---AAAA----------PAAEKKK-EEXESKKEESESDDEDMGFGLFD 127
1.......10........20........30........40........50........60........70........80........90........100.......110.......120......

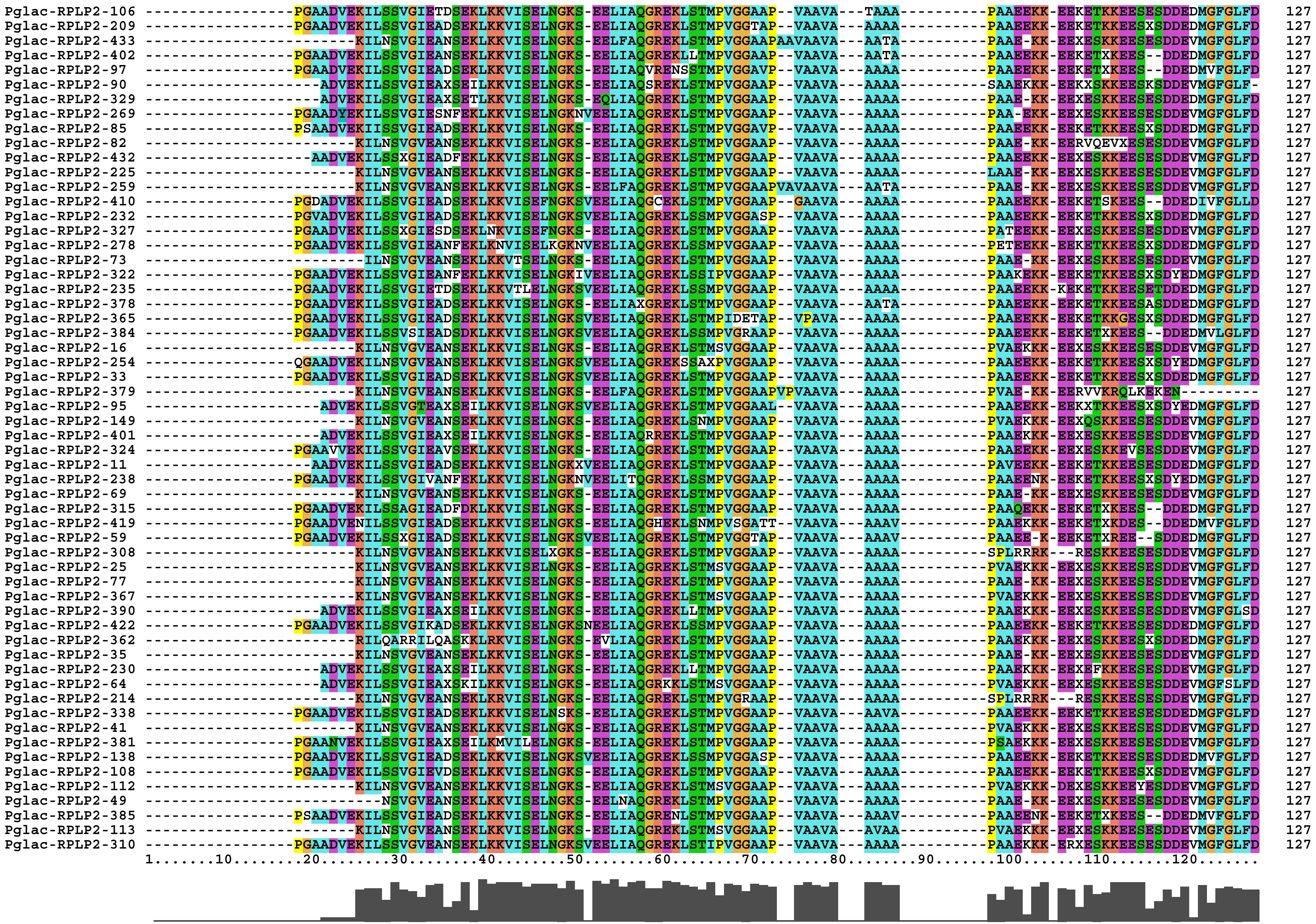
Pgiac-RPLP2-106 127
Pgiac-RPLP2-209 127
Pgiac-RPLP2-433 127
Pgiac-RPLP2-402 127
Pgiac-RPLP2-97 127
Pgiac-RPLP2-90 127
Pgiac-RPLP2-329 127
Pgiac-RPLP2-269 127
Pgiac-RPLP2-85 127
Pgiac-RPLP2-82 127
Pgiac-RPLP2-432 127
Pgiac-RPLP2-225 127
Pgiac-RPLP2-259 127
Pgiac-RPLP2-410 127
Pgiac-RPLP2-232 127
Pgiac-RPLP2-327 127
Pgiac-RPLP2-278 127
Pgiac-RPLP2-73 127
Pgiac-RPLP2-322 127
Pgiac-RPLP2-235 127
Pgiac-RPLP2-378 127
Pgiac-RPLP2-365 127
Pgiac-RPLP2-384 127
Pgiac-RPLP2-16 127
Pgiac-RPLP2-254 127
Pgiac-RPLP2-33 127
Pgiac-RPLP2-379 127
Pgiac-RPLP2-95 127
Pgiac-RPLP2-149 127
Pgiac-RPLP2-401 127
Pgiac-RPLP2-324 127
Pgiac-RPLP2-11 127
Pgiac-RPLP2-238 127
Pgiac-RPLP2-69 127
Pgiac-RPLP2-315 127
Pgiac-RPLP2-419 127
Pgiac-RPLP2-59 127
Pgiac-RPLP2-308 127
Pgiac-RPLP2-25 127
Pgiac-RPLP2-77 127
Pgiac-RPLP2-367 127
Pgiac-RPLP2-390 127
Pgiac-RPLP2-422 127
Pgiac-RPLP2-362 127
Pgiac-RPLP2-35 127
Pgiac-RPLP2-230 127
Pgiac-RPLP2-64 127
Pgiac-RPLP2-214 127
Pgiac-RPLP2-338 127
Pgiac-RPLP2-41 127
Pgiac-RPLP2-381 127
Pgiac-RPLP2-138 127
Pgiac-RPLP2-108 127
Pgiac-RPLP2-112 127
Pgiac-RPLP2-49 127
Pgiac-RPLP2-385 127
Pgiac-RPLP2-113 127
Pgiac-RPLP2-310 127
1.......10........20........30........40........50........60........70........80........90........100.......110.......120.......

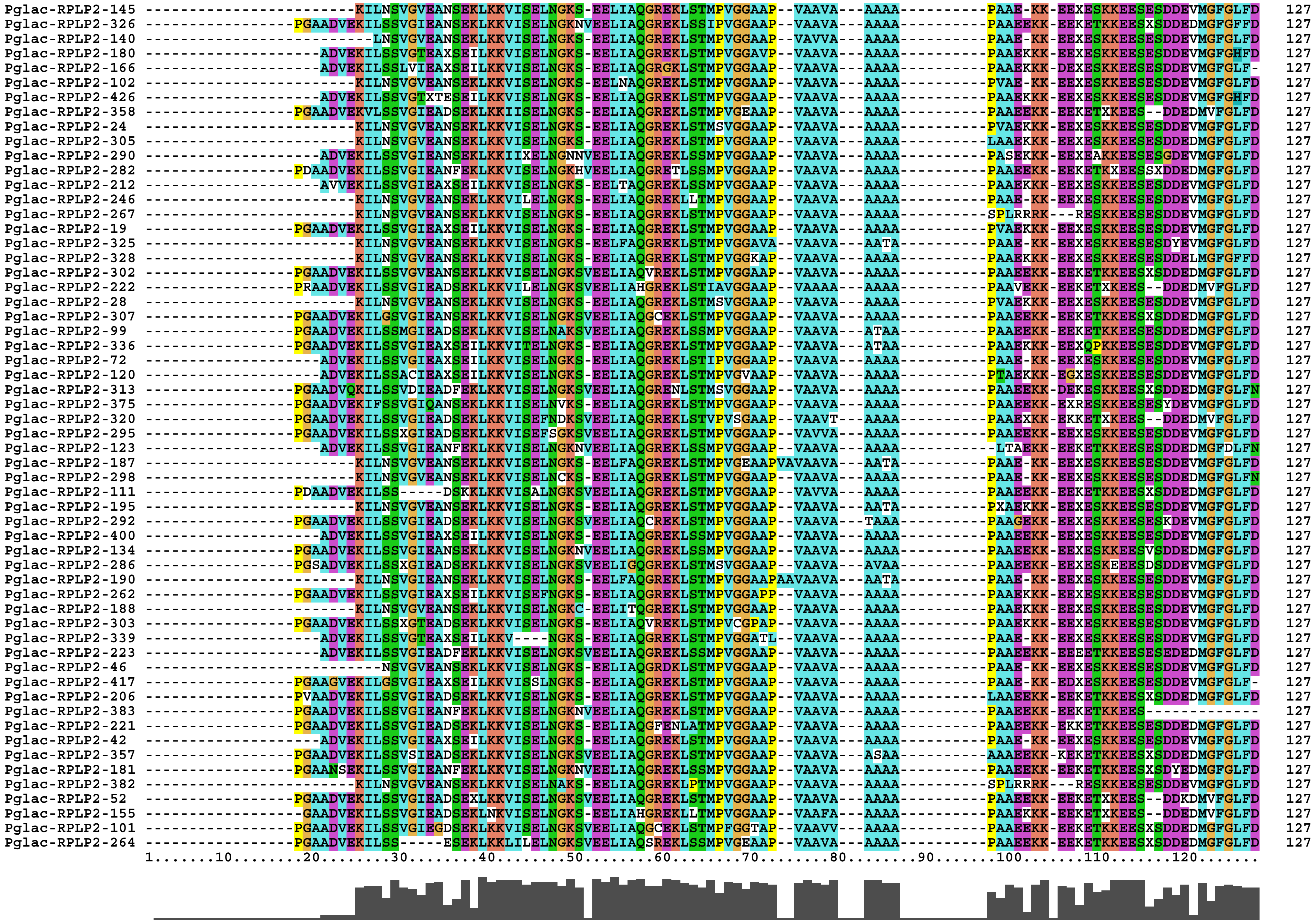
Pglac-RPLP2-145 ------------------------KILNSVGVEANSEKLKKVISELNGKS-EELIAQGREKLSTMPVGGAAP--VAAVA---AAAA----------PAAE-KK-EEXESKKEESESDDEVMGFGLFD 127
Pglac-RPLP2-326 -----------------PGAADVEKILSSVGIEANSEKLKKVISELNGKNVEELIAQGREKLSSIPVGGAAP--VAAVA---AAAA----------PAAEEKK-EEKETKKEESXSDDEDMGFGFFD 127
Pglac-RPLP2-140 --------------------------LNSVGVEANSEKLKKVISELNGKS-EELIAQGREKLSTMPVGGAAP--VAVVA---AAAA----------PAAE-KK-EEXESKKEESESDDEVMGFGLFD 127
Pglac-RPLP2-180 ---------------------ADVEKILSSVGTEAXSEILKKVISELNGKS-EELIAQGREKLSTMPVGGAVP--VAAVA---AAAA----------PAAEKKK-EEXESKKEESESDDEVMGFGHFD 127
Pglac-RPLP2-166 ---------------------ADVEKILSSLVIEAXSEILKKVISELNGKS-EELIAQGRGKLSTMPVGGAAP--VAAVA---AAAA----------PAAEKKK-DEXESKKEESESDDEVMGFGLF- 127
Pglac-RPLP2-102 ------------------------KILNSVGVEANSEKLKKVISELNGKS-EELNAQGREKLSTMPVGGAAP--VAAVA---AAAA----------PVAE-KK-EEXESKKEESESDDEVMGFGLFD 127
Pglac-RPLP2-426 ---------------------ADVEKILSSVGTXTESEILKKVISELNGKS-EELIAQGREKLSTMPVGGAAP--VAAVA---AAAA----------PAAEKKK-EEXESKKEESESDDEVMGFGHFD 127
Pglac-RPLP2-358 -----------------PGAADVEKVLSSVGIEADSEKLKKIISELNGKS-EELIAQGREKLSTMPVGEAAP--VAAVA---AAAA----------PAAEEKK-EEKETXKEES--DDEDMVFGLFD 127
Pglac-RPLP2-24 ------------------------KILNSVGVEANSEKLKKVISELNGKS-EELIAQGREKLSTMSVGGAAP--VAAVA---AAAA----------PVAEKKK-EEXESKKEESESDDEVMGFGLFD 127
Pglac-RPLP2-305 ------------------------KILNSVGVEANSEKLKKVISELNGKS-EELIAQGREKLSTMPVGGAAP--VAAVA---AAAA----------LAAEKKK-EEXESKKEESESDDEDMGFGLFD 127
Pglac-RPLP2-290 ---------------------ADVEKILSSVGIEANSEKLKKIIXELNGNNVEELIAQGREKLSSMPVGGAAP--VAAVA---AAAA----------PASEKKK-EEXEAKKEESESGDEVMGFGLFD 127
Pglac-RPLP2-282 -----------------PDAADVEKILSSVGIEANFEKLKKVISELNGKHVEELIAQGRETLSSMPVGGAAP--VAAVA---AAAA----------PAAEEKK-EEKETKXEESSXDDEDMGFGLFD 127
Pglac-RPLP2-212 ---------------------AVVEKILSSVGIEAXSEILKKVISELNGKS-EELTAQGREKLSSMPVGGAAP--VAAVA---AAAA----------PAAEKKK-EEXESKKEESESDDEVMGFGLFD 127
Pglac-RPLP2-246 ------------------------KILNSVGVEANSEKLKKVILELNGKS-EELIAQGREKLLTMPVGGAAP--VAAVA---AAAA----------PAAE-KK-EEXESKKEESESDDEVMGFGLFD 127
Pglac-RPLP2-267 ------------------------KILNSVGVEANSEKLKKVISELNGKS-EELIAQGREKLSTMPVGGAAP--VAAVA---AAAA----------SPLRRRK---RESKKEESESDDEVMGFGLFD 127
Pglac-RPLP2-19 -----------------PGAADVEKILSSVGIEAXSEILKKVISELNGKS-EELIAQGREKLSTMPVGGAAP--VAAVA---AAAA----------PVAEKKK-EEXESKKEESESDDEVMGFGLFD 127
Pglac-RPLP2-325 ------------------------KILNSVGVEANSEKLKKVISELNGKS-EELFAQGREKLSTMPVGGAVA--VAAVA---AATA----------PAAE-KK-EEXESKKEESESDYEVMGFGLFD 127
Pglac-RPLP2-328 ------------------------KILNSVGVEANSEKLKKVISELNGKS-EELIAQGREKLSTMPVGGKAP--VAAVA---AAAA----------PAAEKKK-EEXESKKEESESDDELMGFGFFD 127
Pglac-RPLP2-302 -----------------PGAADVEKILSSVGVEANSEKLKKVISELNGKSVEELIAQVREKLSTMPVGGAAP--VAAVA---AAAA----------PAAEEKK-EEKETKKEESXSDDEDMGFGLFD 127
Pglac-RPLP2-222 -----------------PRAADVEKILSSVGIEADSEKLKKVILELNGKSVEELIAHGREKLSTIAVGGAAP--VAAAA---AAAA----------PAAVEKK-EEKETXKEES--DDEDMVFGLFD 127
Pglac-RPLP2-28 ------------------------KILNSVGVEANSEKLKKVISELNGKS-EELIAQGREKLSTMSVGGAAP--VAAVA---AAAA----------PVAEKKK-EEXESKKEESESDDEVMGFGLFD 127
Pglac-RPLP2-307 -----------------PGAADVEKILGSVGIEANSEKLKKVISELNGKSVEELIAQGCEKLSTMPVGGAAP--VAAVA---AAAA----------PAAEEKK-EEKETKKEESXSDDEDMGFGLFD 127
Pglac-RPLP2-99 -----------------PGAADVEKILSSMGIEADSEKLKKVISELNAKSVEELIAQGREKLSSMPVGGAAP--VAAVA---ATAA----------PAAEEKK-EEKETKKEESESDDEDMGFGLFD 127
Pglac-RPLP2-336 -----------------PGAADVEKILSSVGIEAXSEILKKVITELNGKS-EELIAQGREKLSTMPVGGAAP--VAAVA---ATAA----------PAAEKKK-EEXQPKKEESESDDEVMGFGLFD 127
Pglac-RPLP2-72 ---------------------ADVEKILSSVGIEAXSEILKKVISELNGKS-EELIAQGREKLSTIPVGGAAP--VAAVA---AAAA----------PAAE-KK-EEXESKKEESESDDEVMGFGLFD 127
Pglac-RPLP2-120 ---------------------ADVEKILSSACIEAXSEILKKVISELNGKS-EELIAQGREKLSTMPVGVAAP--VAAVA---AAAA----------PTAEKKK-EGXESKKEESESDDEVMGFGLFD 127
Pglac-RPLP2-313 -----------------PGAADVQKILSSVDIEADFEKLKKVISELNGKSVEELIAQGRENLSTMSVGGAAP--VAAVA---AAAA----------PAAEEKK-DEKESKKEESXSDDEDMGFGLFN 127
Pglac-RPLP2-375 -----------------PGAADVEKIFSSVGIQANSEKLKKIISELNVKS-EELIAQGREKLSTMPVGGAAP--VAAVA---AAAA----------PAAEEKK-EXRESKKEESESYDEVMGFGLFD 127
Pglac-RPLP2-320 -----------------PGAADVEKILSSVGIEADSEKLKKVISEFNDKSVEELIAQGREKLSTVPVSGAAP--VAAVT---AAAA----------PAAEXKK-EKKETXKEES--DDEDMVFGLFD 127
Pglac-RPLP2-295 -----------------PGAADVEKILSSXGIEADSEKLKKVISEFSGKSVEELIAQGREKLSTMPVGGAAP--VAVVA---AAAA----------PAAEEKK-EEXESKKEESESDDEVMGFGLFD 127
Pglac-RPLP2-123 ---------------------ADVEKILSSVGIEANFEKLKKVISELNGKNVEELIAQGREKLSSMPVGGAAP--VAAVA---AAAA-----------LTAEKK-EEKETKKEESESDDEDMGFDLFN 127
Pglac-RPLP2-187 ------------------------KILNSVGVEANSEKLKKVISELNGKS-EELFAQGREKLSTMPVGEAAPVAVAAVA---AATA----------PAAE-KK-EEXESKKEESESDDEVMGFGLFD 127
Pglac-RPLP2-298 ------------------------KILNSVGVEANSEKLKKVISELNCKS-EELIAQGREKLSTMPVGGAAP--VAAVA---AAAA----------PAAE-KK-EEXESKKEESESDDEDMGFGLFN 127
Pglac-RPLP2-111 -----------------PDAADVEKILSS-----DSKKLKKVISALNGKSVEELIAQGREKLSTMPVGGAAP--VAVVA---AAAA----------PAAEEKK-EEKETKKEESXSDDEDMGFGLFD 127
Pglac-RPLP2-195 ------------------------KILNSVGVEANSEKLKKVISELNGKS-EELIAQGREKLSTMPVGGAAP--VAAVA---AATA----------PXAEKKK-EEXESKKEESESDDEVMGFGLFD 127
Pglac-RPLP2-292 -----------------PGAADVEKILSSVGIEADSEKLKKVISELNGKSVEELIAQCREKLSTMPVGGAAP--VAAVA---TAAA----------PAAGEKK-EEXESKKEESESKDEVMGFGLFD 127
Pglac-RPLP2-400 ---------------------ADVEKILSSVGIEAXSEILKKVISELNGKS-EELIAQGREKLSSMPVGGAAP--VAAVA---AAAA----------PAAEEKK-EEXESKKEESESDDEVMGFGLFD 127
Pglac-RPLP2-134 -----------------PGAADVEKILSSVGIEANSEKLKKVISELNGKNVEELIAQGREKLSSMPVGGAAP--VAAVA---AAAA----------PAAEEKK-EEXESKKEESVSDDEDMGFGLFD 127
Pglac-RPLP2-286 -----------------PGSADVEKILSSXGIEADSEKLKKVISELNGKSVEELIGQGREKLSTMSVGGAAP--VAAVA---AVAA----------PAAEEKK-EEXESKEEESDSDDEVMGFGLFD 127
Pglac-RPLP2-190 ------------------------KILNSVGIEANSEKLKKVISELNGKS-EELFAQGREKLSTMPVGGAAPAAVAAVA---AATA----------PAAE-KK-EEXESKKEESESDDEVMGFGLFD 127
Pglac-RPLP2-262 -----------------PGAADVEKILSSVGIEAXSEILKKVISEFNGKS-EELIAQGREKLSTMPVGGAPP--VAAVA---AAAA----------PAAEKKK-EEXESKKEESESDDEVMGFGLFD 127
Pglac-RPLP2-188 ------------------------KILNSVGVEANSEKLKKVISELNGKC-EELITQGREKLSTMPVGGAAP--VAAVA---AAAA----------PAAEKKK-EEXESKKEESESDDEVMGFGLFD 127
Pglac-RPLP2-303 -----------------PGAADVEKILSSXGTEADSEKLKKVISELNGKS-EELIAQVREKLSTMPVCGPAP--VAAVA---AAAA----------PAAEKKK-EEXESKKEESESDDEVMGFGLFD 127
Pglac-RPLP2-339 ---------------------ADVEKILSSVGTEAXSEILKKV----NGKS-EELIAQGREKLSTMPVGGATL--VAAVA---AAAA----------PAAE-KK-EEXESKKEESESDDEVMGFGLFD 127
Pglac-RPLP2-223 ---------------------ADVEKILSSVGIEADFEKLKKVISELNGKSVEELIAQGREKLSSMPVGGAAP--VAAVA---AAAA----------PAAEEKK-EEEETKKEESESEDEDMGFGLFD 127
Pglac-RPLP2-46 ---------------------------NSVGVEANSEKLKKVISELNGKS-EELIAQGRDKLSTMPVGGAAP--VAAVA---AAAA----------PAAE-KK-EEXESKKEESESDDEVMGFGLFD 127
Pglac-RPLP2-417 -----------------PGAAGVEKILGSVGIEAXSEILKKVISSLNGKS-EELIAQGREKLSTMPVGGAAP--VAAVA---AAAA----------PAAE-KK-EDXESKKEESESDDEVMGFGLF- 127
Pglac-RPLP2-206 -----------------PVAADVEKILSSVGIEADSEKLKKVISELNGKS-EELIAQGREKLSTMPVGGAAP--VAAVA---AAAA----------LAAEEKK-EEKETKKEESXSDDEDMGFGLFD 127
Pglac-RPLP2-383 -----------------PGAADVEKILSSVGIEANFEKLKKVISELNGKNVEELIAQGREKLSTMPVGGAAP--VAAVA---AAAA----------PAAEEKK-EEKETKKEES------------- 127
Pglac-RPLP2-221 -----------------PGAADVEKILSSVGIEADSEKLKKVISELNGKS-EELIAQGFENLATMPVGGAAP--VAAVA---AAAA----------PAAEEKK-EKKETKKEESESDDEDMGFGLFD 127
Pglac-RPLP2-42 ---------------------ADVEKILSSVGIEAXSEILKKVISELNGKS-EELIAQGREKLSTMPVGGAAP--VAAVA---AAAA----------PAAE-KK-EEXESKKEESESDDEVMGFGLFD 127
Pglac-RPLP2-357 -----------------PGAADVEKILSSVSIEADSEKLKKVISELNGKSVEELIAQGREKLSTMPVGGAAP--VAAVA---ASAA----------AAAEEKK-KEKETKKEESXSDDEDMGFGLFD 127
Pglac-RPLP2-181 -----------------PGAANSEKILSSVGIEANFEKLKKVISELNGKNVEELIAQGREKLSSMPVGGAAP--VAAVA---AAAA----------PAAEEKK-EEKETKKEESXSDYEDMGFGLFD 127
Pglac-RPLP2-382 ------------------------KILNSVGVEANSEKLKKVISELNAKS-EELIAQGREKLPTMPVGGAAP--VAAVA---AAAA----------SPLRRRK---RESKKEESESDDEVMGFGLFD 127
Pglac-RPLP2-52 -----------------PGAADVEKILSSVGIEADSEXLKKVISELNGKSVEELIAQGREKLSTMPVGGAAP--VAAVA---AAAA----------PAAEEKK-EEKETXKEES--DDKDMVFGLFD 127
Pglac-RPLP2-155 ------------------GAADVEKILSSVGIEADSEKLNKVISELNGKS-EELIAHGREKLLTMPVGGAAP--VAAFA---AAAA----------PAAEKKK-EEKETXKEES--DDEDMVFGLFD 127
Pglac-RPLP2-101 -----------------PGAADVEKILSSVGIEGDSEKLKKVISELNGKSVEELIAQGCEKLSTMPFGGTAP--VAAVV---AAAA----------PAAEEKK-EEKETKKEESXSDDEDMGFGLFD 127
Pglac-RPLP2-264 -----------------PGAADVEKILSS-----ESEKLKKLILELNGKSVEELIAQSREKLSSMPVGEAAP--VAAVA---AAAA----------PAAEEKK-EEKETKKEESXSDDEDMGFGLFD 127
1.......10........20........30........40........50........60........70........80........90.......100.......110.......120.......

```
Pglac-RPLP2-363 -----------------PGAADVEKIFSSVCIESDSEKLKKVISEFNGKS-EELIAQGREKLSTMPVGGAAP--VAAVA---AAAA----------PAAE-KK-EEKETKKEESXSDDEDMGFGLFD  127
Pglac-RPLP2-257 ------------------------KILKSVGVEANSEKLKKVISELNGKS-EELIAQGREKLSTMPVGGAAP--VAAVA---AATA----------PXAEKKK-EEXESKKEESESDDEVMGFGLFD  127
Pglac-RPLP2-186 -----------------PGAADVEKILSSVGIEADSEKLKKVISELNDKSVEELIAQGREKLSSMPFDGAAP--VAAVA---AAAA----------PAAEEKK-EEKETXKEES--DDEDMVFGLFD  127
Pglac-RPLP2-165 -----------------PGAVDVEKILSSVGIEAXSEILKKVISELNGKS-EELIAQGREKLSTMPVGGAAP--VAAVA---AAAA----------PAAEEKK-EEKETKKEESXSDDEDMGFDLFD  127
Pglac-RPLP2-169 ------------------------KILNSVGVEANSEKLKKVISELNGKS-EELIAQGREKLSTMSVGGAAP--VAAVA---AAAA----------PVAEKKK-EEXESKKEESESDDEVMGFGLFD  127
Pglac-RPLP2-398 ------------------GAADVEKILSSVGIEAXSEILKKFISELNGKS-EELIAQGREKLSTMPVGGVAP--VAAVA---AAAA----------PAAE-RK-EEXESKKEESESDDEVMGFGLFD  127
Pglac-RPLP2-127 -----------------PGAAHVEKILSSVGMEAXSEILKKVISELNGKS-EELIAQVREKLSTMPVGGAAP--VAAVA---AAAA----------PAAEKKK-EEXESKKKESESDDEVMGFGLFD  127
Pglac-RPLP2-270 ------------------SVSDVEKILSSVGIEANFEKLKKVTSEFNGKNVEELIAQGREKLSSMPVGGAAP--VAAVA---AAEA----------LAAEEKK-EEKETKKEESXSDYEDMGFGLFD  127
Pglac-RPLP2-393 -----------------PGAADVEKILSSVGIEAXSEILKKVISELNGKS-EELIAQGREKLLTMPVGGAAP--VAAVA---AAAA----------PAAEKKK-EEXESKKEESESDDEVMGFGLFD  127
Pglac-RPLP2-376 -----------------PGAADVEKILSSVGFEADSKKLKKVISELNGKSDEELIAQGREKLSSMPVGGAAP--VAAVA---AAAA----------PAA-EKK-EEKETXKEES--DDEDMVFGLFD  127
Pglac-RPLP2-10  ------------------------KILNSVGVEANSEKLKKVISELNGKS-EELIAQGREKLSTMSVGGAAP--VAAVA---AVAA----------PVAEKKK-EEXESKKEESESDDEVMGFGLFD  127
Pglac-RPLP2-7   -----------------PGAADVEKILSSVGIEVDSEKLKKVISELNGKSVEELIAQGREKLSSMPVGGASP--VAAVA---AAAA----------PAAEEKK-EEKETKKEESESDDEDMSLGLFD  127
Pglac-RPLP2-20  ---------------------------NSVGVEANSEKLKKVISELNGKS-EELIAQGREKLSTMPVGGAAP--VAAVA---AAAA----------PAAE-KK-EEXESKKEESESDDEVMGFGLFD  127
Pglac-RPLP2-312 -----------------PGAADVEKILSSVGIEADSEKLKKVISXLNGKSVEELIAQGSEKLSSMPVGGAAP--VAAVA---AAAA----------PAAKENK-EEKETKKEES--DDEDMGFGLFD  127
Pglac-RPLP2-311 --------------------ADVDKILSSVGIEAXSEILKKVISELNCKS-EELIAQGREKLSTMPVGGAAP--VAAVA---AAAA----------SPLRRRK---RESKKEESESDDEVMGFGLFD  127
Pglac-RPLP2-27  ------------------------KILNSVGVEANSEKLKKVISELNGKS-EELIAQGREKLSTMSVGGAAP--VAAVA---AAAA----------PVAEKKK-EEXESKKEESESDDEVMGFGLFD  127
Pglac-RPLP2-204 -------------------------ILNSVGVEASSEKLKKVISEL--KS-EELIAQGREKLSTMPVGGAAP--VAAVA---AATA----------PXAEKKK-EEXESKKEESESDDEVMGFGLFD  127
Pglac-RPLP2-348 ------------------------KILNSVGVEANSEKLKKVISELNGKS-EELFAQGREKLSTMPVGGAAPVAVAAVA---AATA----------PXAEKKK-EEXESKKEESESDDEVMGFGLFD  127
Pglac-RPLP2-179 ------------------------KILNSVGVEANFEKLKKVISELNGKS-EELIAQGREKLSTMSVGGAAP--VAAVA---AVAA----------PVAEKKK-EEXESKKEESESDDEVMGFGLFD  127
Pglac-RPLP2-265 -----------------PGAADVEKILSSVGIEAXSEILKKVISELNGKS-EELIAQGREKLSTMPVGGAAP--VAAVA---AAAA----------PAAEEKK-EEKETKKEESXSDDEDMGFGLFD  127
Pglac-RPLP2-211 -----------------PGAADVEKILSSVGIEADSEKLKKVISELNGKS-EELIAQGREKLSTIPVGGAAS--VAAVA---AAAA----------PAAEEKK-EEKETKKEESXSDDEDMGFGLFD  127
Pglac-RPLP2-420 -----------------PGAADVENILSSVGIEADSEKLKKVISELNGKS-EELIAQGREKLSNMPVSGAAP--VAAVA---AAAV----------PAAEEKK-EEKETXKDES--DDEDMVFGLFD  127
Pglac-RPLP2-12  ---------------------------NSVGVEANSEKLKKVISELNGKS-EELIAQGREKLSTMPVGGAAP--VAAVA---AAAA----------PAAE-KK-EEXESKKEESESDDEVMGFGLFD  127
Pglac-RPLP2-334 -----------------PGAADVKKILSSVGIEADSEKLKKVISELNGKS-EELIAQGREKLSTMPVGGAAP--VAAVA---AAEA----------PAA-EKK-EEKETXKEES--DDEDMVFGLFD  127
Pglac-RPLP2-159 ------------------SSADVEKILSSVGIEAXSEILKKVISELNGKS-EELIAQGREKLSTMPVGGAAP--VAAVA---AAAA----------PAAEKKK-EERVQEGIXSESDDEDMGFGLFD  127
Pglac-RPLP2-394 -----------------PGAADVEKILSSVGIEADSEKLKKVISELNGKK-EELIVQGREKLSTIPVGGAAP--VAAVA---AAVA----------PAAEEKK-EEKETXKEKS--DDEDMVFGLFD  127
Pglac-RPLP2-319 -----------------PGTADVEKILSSVGIEADSEKLKKVISELNGKS-KELIAQGREKLSTMPVGGAAP--VDAVA---AAAA----------PAA-EKK-EEKVTXKEE--SDDEDMVFGLFD  127
Pglac-RPLP2-333 -----------------PGAPDVEKILSSVGIEADSEKLKKVISELNGKS-EELIAQGREKSSTMPVGGAAP--VAAVA---AAAA----------PAAEEKK-EEKETKKEESXSDDGDMGFGLFD  127
Pglac-RPLP2-421 -----------------PGAADVENILSSVGIEADSEKLKMVISELNGKS-EELIAQGHEKLTNMPVSGAAP--VAAVA---AAAV----------PAAEEKK-EEKETXKDES--DDEDMVFGLFD  127
Pglac-RPLP2-380 -----------------PGAADVEKILSSVGIEAXSEILKKVISELNGKS-EELIAQGREKLSTMPVGGAAS--VAAVA---TAAA----------PAAEKKK-EEXESKKEESXSDDEDMGFGLFD  127
Pglac-RPLP2-217 --------------------ADVEKILSSVGIEAXSEILKKVISELNGKS-EELIAQGREKLSTMPVGGTAP--VAAVA---AAAA----------PAAE-KK-EERESKKEESESDDEVMGFGLFD  127
Pglac-RPLP2-103 --------------------ADVEKILSSVGIEANFEKLKKVISELNGKS-EELIAQGREKLSTLPVGGAAP--VAAVA---AAAA----------PAA-EKK-EEXESKKEESESDDEVMDFGLFD  127
Pglac-RPLP2-323 ------------------------KILNSVGVEANSEKLKKVISELNGKS-EELITQGREKLSTMPVGGAAP--VAAVA---AAAA----------PVAEKKK-EEXESKKEESESDDEDMGFGLFD  127
Pglac-RPLP2-240 ------------------------------GILQASKKLRKVISELNGKS-EELIAQGREKLSTMPVGGAAP--VAAAAAPPAAAT----------VPAAEKK-EEXESKKEESESDDEVMGFGLFD  127
Pglac-RPLP2-87  -----------------PGAADVEKILSSVGIEADSEKLKKVISELNGKS-EELIAQGREKLSTMPVGGAAP--VAAVA---TAAA----------PAAEEKK-EEKETKKEESXSDDEDMGFSLFD  127
Pglac-RPLP2-335 ------------------GAADXEKILSSVGIEAXSEILKKVISELNGKS-EELIAXGREKLSTMPVGGAAP--VAAVX---AAAA----------PAAEKRK---RESKKEESESDDEVMGFGLFD  127
Pglac-RPLP2-80  ------------------------KILNSVGVEANSEKLKKVISELNGKS-EELIAQGREKLSTMSVGGAAP--VAAVA---AAAA----------PVAEKKK-EEXESKKEESESDDEVMGFGLFD  127
Pglac-RPLP2-396 --------------------ADVEKILSSVGIEAXSEILKKVISELNGKS-EXLIAQGREKLSTMPVGGAAP--WAAVA---AAAA----------PAAE-KK-EEXESKKEKSESDDEVMGFGLFN  127
Pglac-RPLP2-291 -----------------PGAADVEKILSSVGIEANFKKLKKVISELNGKNVEELIAQGREKLSSMPVGGAAP--VAAVA---AAAA----------PAAEEKK-EEKETKKEESXSDYEDMGFGLFD  127
Pglac-RPLP2-248 -----------------PGAADVEKIISSVDIEADSEKLKKVISELNGKSIEELIAQGREKLSSMPVDGAAP--VAAVV---AAAA----------PVAEEKK-EEKETKKEESESDDEDMGFGLFD  127
Pglac-RPLP2-424 -----------------PGAADVEKILSSXGTEADSEKLKKVISELNGKS-EQLIAQGREKLSTMPVGGAAP--VAAVA---AAAA----------PAAEKKK-EEXESKKEESESDDEVMGFGLFD  127
Pglac-RPLP2-412 -----------------PGAADVEKILSSVGIEANFEKLKKVISELNGENVEELIAQGRENLSSMPVGGAAP--VAVVA---AAAA----------PAAEEKK-EEKETKKEESXSDYEDMGFGLFD  127
Pglac-RPLP2-360 ------------------------KILNSVGVEANSEKLKKVISELNGKS-EELFAQGREKLSTMPVGGAAPVPVAAVA---AAAA----------PVAE-KK-EEXESKKEESESDDEVMGFGLLD  127
Pglac-RPLP2-126 ------------------------KILNSVGVEANSEKLKKVISELNGKS-EELIAQGREKLSTMPVGGAAP--VAAVA---AAAA----------PAAE-KK-EEXESKKEESESDDEVMGFGLFD  127
Pglac-RPLP2-219 --------------------ADVEKIPSSVGIEAXSEILKKVISELNGKS-EELIAQGREKLSTMPVGGAAP--VAAVA---AAAA----------PAAE-KK-EEXESKKEESESDDEVMGFGLFD  127
Pglac-RPLP2-168 -----------------PRAAAVEKILSSVVIEADSEKLKKVISELNGKSVEELIAQAREKLSSMPVGGAAP--VAAVA---AAVA----------PAAEEKK-EEKETKKEESESDDEDMGFGLFD  127
Pglac-RPLP2-203 ------------------GAADVEKILSSVGIEADSEKLKKVISELNGKSVEELIAQGREKLSSMPVGGAAP--VAAVA---AAAA----------PAAEEKK-KEKETKKEESEADDEDMGFGLFD  127
Pglac-RPLP2-239 ------------------------KILNSVGVEANSEKLKKVISELNGKS-EELFAQGREKLSTMPVGGAAPVPVAAVA---AAAA----------PVAE-KK-EEXESKMEESESDDEVMGFGLFD  127
Pglac-RPLP2-344 --------------------ADVEKILSSVGIEAXSEILKKVISELNGKS-EELIAQGREKLSTMPVGGTAP--VAAVA---AAAA----------PAAEKKK-EEXESKKEESESDDEDMGFGLFD  127
Pglac-RPLP2-227 -----------------PGAADIEKILSSVGIEADSEKLKKVISELNGKS-EELIAQGREKLSTMPVSGAAP--VAAVA---AAAA----------PAAEEKK-EEKETXKEES--DNEDMVFGLFD  127
Pglac-RPLP2-144 ------------------------KILNSVGVEANSEKLKKVISELNGKS-EELIAQGREKLSTMPVSGAAP--VAAVA---AAAA----------PAAE-KK-EEXESKKEESESEDEVMCFGLFD  127
Pglac-RPLP2-287 --------------------ADVEKILSSVGTEAXSEILMKVISELNGKS-EELIAQGREKLSTMPVGGAAP--VAAVA---AAAT----------LAAEKKK-EEXESKKEESESDNEVMGFGLFN  127
Pglac-RPLP2-405 --------------------ADVEKILSSLGIEAXSEILKKVISELNGKS-EELIAQGREKLSTMPVSGAAP--VAAVA---AAAV----------LVAKKKK-EEXESKKEESESDDEVMGFGLFD  127
Pglac-RPLP2-386 -----------------PGAANVEKILSSVGIEADSEKLKKVISELNGKSVEELIAQGREKLSSMPXGEAAP--VAAVA---AAAA----------PAAEEKK-EEKETKKEESESDDEDMGFGLFD  127
Pglac-RPLP2-98  -----------------PGAADVEKILSSXGIEADSEKLKKVISELNSKSVEELIAQGREKLSTMPVGGAVP--VAAVA---AAAA----------PAAEEKK-EEXESKKEESESDDEVMGFGLFD  127
Pglac-RPLP2-13  -----------------PGAADVEKILSSVGIEADSEKLKKVISELNGKS-EELIAQGREKLSTMPVGGAAP--VAAIA---AAAA----------PAAEEKK-EEXESKKEESESDDEVMGFGLFD  127
Pglac-RPLP2-399 -----------------PGAADVEKILSSVGIEADSEKLKKVISELNGKS-EELIAQGREKLSTMPVGGAAP--VAAVA---AAAA----------PEAEEKK-EEKETKKEESXSDDEDMGFGLFD  127
Pglac-RPLP2-164 -----------------PGAADVEKILSSVGIEANSEKLKKVISELNGKNVEELIAQGREKLSSMPVGGEAP--VAAVA---AAAA----------PAAEEKK-EEKETKKEESXSDYEDMGFGLFD  127
                1.......10........20........30........40........50........60........70........80........90........100.......110.......120.......
```

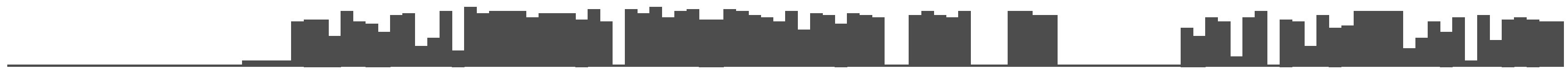

```
Pglac-RPLP2-139 ------------------------KILNSVGVEANSENLKNVISELNGKS-EELIAQGREKLSTMSVGGAAP--VAAVA---AAAA----------PVAEKKK-EEXESKKEESESDDEVMGFGLFD 127
Pglac-RPLP2-32  ------------------------KILNSVGVEANSEKLKKVISELNGKS-EELIAQGREKLSTMSVGGAAP--VAAVA---AAAA----------PVAEKKK-EEXESKKEESESDDEVMGFGLFD 127
Pglac-RPLP2-234 --------------------ADVEKILSSLGIDAXSEILKKVISELNGKS-EELIAQGREKLSTMPVGGAAP--VAAVA---AAAA----------PAAEKKK-EEXESKKEESESDDEVMGFGLFD 127
Pglac-RPLP2-143 ------------------------KILNSVGVEANSEKLKKVISELNGKS-EELIAQGREKLSTMPVGGAAPVAVAAVA---AAAA----------PAAEKKK--EXESKKEESESDDEVMGFGLFD 127
Pglac-RPLP2-92  ------------------------KILNSVGVEANSEKLKKVISELNGKS-EELIAQGREKLSTMPVGGAAP--VAAVT---AAAA----------PAAE-KK-EEXESKKEESESDDEVMGFGLFD 127
Pglac-RPLP2-21  ----------------------------SVGVEANSEKLKKVISELNGKSVEELIAQGREKLSTMPVGGAAP--VAAVA---AAAA----------PAAEEKK-EEKETKKEESXSDDEDMGFGLFD 127
Pglac-RPLP2-162 ------------------------KILNSVGVEANSEKLKKVISELNGKS-EEVIAQGREKLSTMPVGGAAP--VAAVA---AAAA----------PAAE-KK-EEXESKKEESESDDEVMGFGLFD 127
Pglac-RPLP2-189 ------------------------KILNSVGVEANSEKLKKVISELNGKS-EELIAQGREKLSTMPVGGAAP--VAAVA---AAAA----------PAAEKKK-EEXESKKEESESDDEVMGFGLFD 127
Pglac-RPLP2-237 -----------------PGAADVEKILSSVGXEANFEKLKKVISELNGKNVEELIAQGREKLSSMPVGGAAP--VAAVA---AAAA----------PAGEEKK-EEKETKKEESXSDYEDMGFGLFD 127
Pglac-RPLP2-56  -----------------PGAADVEKILSSVGIEAXSEILKKVITELNGKS-EELIAQGREKLSSMPVGEAAP--VAAVA---AAAA----------PAAEKKK-EEXESKKEESESDDEVMGFGLFD 127
Pglac-RPLP2-389 -------------------SADVEKILSSVGIEANSEKLKKVISELNGKNXEELIAQGREKLSSMPVGGAAP--VAAVA---ATAA----------PAAEEKK-EEKETXKEES--DDEDMVFGLFD 127
Pglac-RPLP2-53  ------------------------------GVEANSEKLKKVISELNDKS-EELIAQGREKLSTMPVGGAAPVAVAAVA---AATA----------PAAE-KK-EEXESKKEESESDDEVMGFGLFD 127
Pglac-RPLP2-351 --------------------ADVEKILSSVGTEAXSEILKKVISELNGKSVEELIAQDREK--XLPVGGAAP--VAAVA---AAAA----------PAAEEKK-EEKETKKEESXSDYEDMGFGLFD 127
Pglac-RPLP2-318 ------------------------KILSPVGIEADSEKLKKVISELNGKNVEELIAQGREKLSTMPVGGAVP--VAAVA---AAAA----------PAAEXKK-EEKETKKEESXSDDEDMGFGLFD 127
Pglac-RPLP2-218 ------------------------KILNSVGDEANSEKLKKVISELNGKS-EELIAQGRKKLSTMPVGGAAPVAVAAVA---AAAA----------PAAE-KK-EEXESKKEESESDDEVMGFGLFD 127
Pglac-RPLP2-371 ------------------------KILNSVGVEANSEKLKKVISELNGKS-EELIAQGREKLSTMPVGGAAPVAVAAVA---AAAA----------PAAE-KK-EEXESKKEESESDDEVMGFGLFD 127
Pglac-RPLP2-306 -----------------PGAADVEKILSSVGIEADSEKLKKVIXELNGKT-EELIAQGREKLSTMPVGGAAP--VAAVA---AAAA----------LSAEEKK-EEXESKKEESESDDEVMGFDLFD 127
Pglac-RPLP2-54  ------------------------KILNSVGVEANSEKLKKVISELNGKS-EELIAQGREKLSTMSVGGAAP--VAAVA---AAAA----------PVAEKKK-EEXESKKEESESDDEVMGFGLFD 127
Pglac-RPLP2-377 ------------------GAADVEKILSSXGIEAVSEKL-KVISELNGKS-EELIAQGREKLSTMPVGGAAP--VAAVA---AAAA----------PAAEEKK-EEXESKKEESESDDEDMGFGLCD 127
Pglac-RPLP2-122 ---------------------------NSVGVEANSEKLKKVISELYGKS-VELIAQGREKLSTMPVGGAAP--VAAVA---AAAA----------PAAE-KK-EEXESKKEESESDDEVMGFGLFD 127
Pglac-RPLP2-216 --------------------------INSVGVEANSEKLKKVISELNGKS-EELIAQGREKLSTMPVGGAAP--VAAVA---AATA----------PAAE-KK-EEXESKKEESESDDEVMGFGLFD 127
Pglac-RPLP2-129 -----------------PGAADVEKILSSVGIEADSEKLKKVISKLNGKSVEELIAQGREKLSTMPVGGAAP--VAAVA---AAAA----------PAAEEKK-EEKETKKEESESDDEDMGFGLFD 127
Pglac-RPLP2-154 --------------------ADVEKILSSVGIEAXSEILKKVISELNGKS-EELIAQGREKLSTMPVGGAAP--VAAVA---AAAA----------PAAEKKK-EEXESKKEESESDDEVMGFGLFD 127
Pglac-RPLP2-89  --------------------ADVEKILSSVGVEAXSEILKKVISELNGKS-EELIAQGREKLSTMPVGGAAP--VAAVA---AAAA----------PAAEKKK-EEXESKKEESESDDEVMGFGLFD 127
Pglac-RPLP2-201 ------------------------KILNSVGVEANSEKLKKVISELNGKS-EELIAQGREKLSTMSVGGAAP--VAAVA---AAAA----------PVAE-KX-EERESKKEESESVDEVVGFGLFD 127
Pglac-RPLP2-31  -----------------PGAADVEKILSSVGIEADSEKLKKVISELNGKSVEELIAQGREKLSSMPVGGAAP--VATVA---AAAA----------PAAEEKK-EEKETKKEESESDDEDMGFGLFD 127
Pglac-RPLP2-263 -----------------PGTADVEKILSSVGIEANSEKLKKVISELTGKSVEELIAQGCEKLSTMPVGGAAP--VAAIA---AAAV----------PAAEEKK-EEKETKKEESXSDDEDMGFGLFD 127
Pglac-RPLP2-132 -----------------PGAADVEKILSSVGIEADSEKLKKVISELNGKS-EELIAQGREKLSTMPVGGAAP--VAAVA---AAAA----------PAAEEKK-EEKETXKEES--DDEDMVFGLFN 127
Pglac-RPLP2-178 ------------------------KILNSVGVEANSEKLKKVISELNGKS-EELIAQGREKLSTMSVGGAAP--VAAVA---AAAA----------PVAEKKK-EEXESKKEESESDDEVMGFGLFD 127
Pglac-RPLP2-91  ------------------------KILNSVGVEANSEKLKKVISELNGKS-EELIAQGREKLSTMPVGGAAP--VAAVA---AATA----------XVAEKKK-EEXESKKEESESDDEVMGFGLFD 127
Pglac-RPLP2-170 --------------------ADVEKILSSVGIEAXSEILKKVISELNGKS-EELIAQGREKLSTMPVGGAAP--VAAVA---AAAA----------PAAE-KK-EEXESKKEESESDDEVMGFG--- 127
Pglac-RPLP2-256 -----------------PGAADVEKILSSVGVEANSEKLKKVISELNGKSVEELIAQGREKLSTIPVGGAAP--VAAVA---GAAA----------PAAEEKK-EEKESKKEESESVDEVMGFGLFD 127
Pglac-RPLP2-142 -----------------PGAADVEKILSSVGIEANFEKLKKVISELNGKNVEELIAQGREKLSSMPVGGAAP--VAAVA---AAAA----------PAAEEKK-EEKETKKEESXSDDEDMGFGLFD 127
Pglac-RPLP2-414 -----------------SGAADVEKILSSVGIEADSEKLKKVISELNGKSVEELIAQGREKLLTIPVGGAAP--VAAVT---AAAA----------PAAEEKK-EEKXSKKEESESDDEVMGFGLFD 127
Pglac-RPLP2-151 ------------------------KILNSVGVEANSEKLKKVISELNGKS-EELIAQGREKLSTMSVGAAAP--VAAVA---AAAA----------PVAEKKK-EEXESKKEESETDDEVMGFGLFD 127
Pglac-RPLP2-273 -----------------PGAADVEKILSSVGIEANSEKLKKVISELNCKNVEELIAQGREKLSSMPDGGAAP--VAAIA---AAAA----------PSAEEKK-EEKETKKEES------------- 127
Pglac-RPLP2-93  -----------------PGAADIEKILSSVGVEANSEKLKKVISELNGKSVEELIAQGREKLSTMPVGGAAP--VAAVA---AAAA----------PAAEE-K-EEKETKKED-------------- 127
Pglac-RPLP2-175 ------------------------KILNSVGVEANSEKLKKVISELNGKS-EELIAQGREKLSTMPVGGAAP--VAAVA---AATA----------PXAEKKK-EEXESKKEESESDDEVMGFGLFD 127
Pglac-RPLP2-128 -----------------PGAADVERILSSVGIEADSEKLKKVISELNDKSVEELIAQGREKLSTMPVGGAAP--VAAVA---AAAA----------PAAEEKK-EEKVTXKEES--DDEDMVFGLFD 127
Pglac-RPLP2-304 -----------------PGAADVEKILSSVGIEADSEKLKKVISELNGKSIEELIAQGREKLSSMPVGGAAP--VAAAA---AAAA----------PAAEEKK-EEKETKKEESEPDDEDMGFGLFD 127
Pglac-RPLP2-76  ------------------------KILNSVGVEANSEKLKKVISELNGKS-EELIAQGREKLSTMSVGGAAP--VAAVA---AAAA----------PVAEKKK-EEXESKKEESESDDEVMGFGLFD 127
Pglac-RPLP2-23  --------------------ADVEKILSSACIEAXSEILKKVISELNGKS-EELIAQGREKLSTMPVGVAAP--VAAVA---AAAA----------PAAEKKK-EGXESKKEESESDDEVMGFGLFD 127
Pglac-RPLP2-196 --------------------ADVEKNLSSVGIEANFEKL-KVISELNGKS-EELIAQGREKLSTMPVGGAAP--VAAVA---AAAA----------PAA-EKK-EEXESKKEESESDDEVMGFGLFD 127
Pglac-RPLP2-395 -----------------PGAADVEKILSSVGIEADSGKLKKVISELNGKSVEELIAQGREKLSSMPVGGAAP--VAAVA---AAAA----------PAAEEKK-EEKETKKEESESDDEETGFGLFD 127
Pglac-RPLP2-137 -----------------PGAADVEKILSSVGIEANSEKL-EVISELNGKNVEELIAQGREKFSSMPVGGAAP--VAAVA---AAAA----------PAAEEKK-EEKETKKEESXSDDEDMGFGLFD 127
Pglac-RPLP2-104 --------------------ADVEKILSSVGIEADSETLKKVISELNGKS-EELIAQGREKLSTMPVGGAAP--VAAVA---AAAA----------PAAEEKK-EEXESKKEESESDDEVMGFGLFD 127
Pglac-RPLP2-173 -----------------PGAADVEKTLSSVGIEADSKKLKKVISELNGKSVEELIAQGRKKLSSMPVGGAVP--VAAVA---AAAV----------PAAEE-K-EEKEIKKEESXSEYEDMGFGLFD 127
Pglac-RPLP2-280 -----------------PGAADVEKILSSVGIEADSEKLKKVISELNGKS-EELIAQGCEKLSTMPVGGAAP--VVAAA---AAAA----------PAAEEKK-EEKETKKEES--DDEDMVSGLFD 127
Pglac-RPLP2-387 --------LAVLGGKASQGAADLEKILSSVGIEAXSEILKKVTSELNGKS-EELFAQRREKLSTMPVGGAAP--VSAVA---AAAA----------PAAEKRK-EEXESKKEESESDDEVMGFGLFD 127
Pglac-RPLP2-343 --------------------ADVEKILRSVGIEANSEKLKKIISELNGKNGEELIAQGREKLSSMPVGGAAP--VAAVA---AAAA----------PAAEKKK-EEXEAKKEESESDDEVMGFGLFD 127
Pglac-RPLP2-174 --------------------ADVEKILSSVGIEAXSEILKKVISELNGKS-EELIAQGREKLSTMSVGGAAP--VAAVA---AAAA----------PVAEKKK-EEXESKKEESESDDEVMGFGLFD 127
Pglac-RPLP2-141 -----------------PGAADVEKILSSXGIEADSEQLKKVISELNGKSVEELIAQGRKKLSTMPVGGAAP--VAAVT---AAAA----------PAAEEKK-EEKETKKEESESDDEDMGFGLFD 127
Pglac-RPLP2-152 -----------------PGAADVEKILSSVGIEANSEKLKKVISELNGKS-EELIAQGRDKSSTTPVGGAAP--VAAVA---AAAA----------PAAEEKK-EEKETKKEESESDDEDMVFGLFD 127
Pglac-RPLP2-354 -----------------TGAADVEKILSSVGIEANSEKLKKVISELNGKSVEELIAQGREKLSTMPVGGAAP--VAXVA---AAAA----------PAAEEKK-EEKETKKEESXSDDEDMGFGLFD 127
Pglac-RPLP2-252 --------------------ADVEKILSSVGIEAXSDILKKVISELNGKS-EELIAQGREKLSTMPVGGAAP--VAAVA---AAAA----------PAAEKKK-EEXESKKEESDSDDEVMGFGHFD 127
Pglac-RPLP2-60  ------------------------KILNSVGVEANSEKLKKVISELNGKS-EELIAQGREKLSTMPVGGAAP--VAAVA---AAAA----------PAAE-KK-EEXESKKEESESDDEVMGFGLFD 127
Pglac-RPLP2-429 -----------------PGAADVEKILSSVGTEADSEKLKKVISELNGKS-EELIAQGREKLSTMPVGGAAS--VAAVA---AAAA----------PAAEEKK-EEKESKXEESESDDEVMGFGLFD 127
Pglac-RPLP2-22  ------------------------KILNSVGVEANSEKLKKVISELNGKS-EELIAQGREKLSTMSVGGAAP--VAAVA---AAAA----------PVAEKKK-EEXESKKEESESDDEVMGFGLFD 127
                1.......10........20........30........40........50........60........70........80........90.......100.......110.......120.......
```

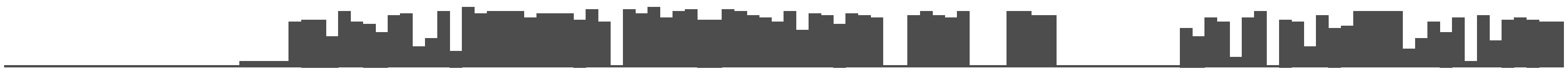

```
Pglac-RPLP2-247 -----------------SGAADVEKILSSVGIEADSEKLKKVISELNGKSVEELIAQGREKLSSMPVDGAAP--VAAVA---AAAA----------PAAEEKK-EEKETKKEESASYDEDMGFGLFD 127
Pglac-RPLP2-68  --------------------ADVEKILSSVGIEANSEKLKKVISELNGKNVEELIAQGREKLSSMPVGGAAP--VAAVA---AAAA----------PAAEEKK-EEKETKKEESXSDYEDMGFGLFD 127
Pglac-RPLP2-114 -----------------PGAADVEKILSSVGIEANSEKLKKVIXELNGKNVEELIAQGREKLSSMPVGGAAP--VAAVA---AAAA----------PTAEEKK-EEKETXNEES--DDEDMVFGLFD 127
Pglac-RPLP2-194 ------------------------KILNSVGVEANSEKLKKVISELNGKS-EELIAQGSEKLSTMPVGGGAP--VAAVA---AATA----------PXLRRKR--KRESKKEESESDDEVMGFGLFD 127
Pglac-RPLP2-285 ------------------------KILNSVGVEANSEKLKKVISELNGKS-EELFAQGREKLSTMPVGGAAPVPVAAVA---AAAA----------PVAE-KK-EEXESKKEESESDDEVMGFGLLD 127
Pglac-RPLP2-131 -----------------PGAADVEKILSSVGIEANSEKLKKVISELNGKNVEXLIAQGREKLSSMPVGGATP--VAAVA---AAAT----------PAAEEKK-EEKETMKEESESDDEDMGFGLFD 127
Pglac-RPLP2-117 ------------------GAANVEKILSSVGIEANSEILKKVISELNGKS-EELIAQGREKLSTMPVGRAAP--VAAVA---AAAA----------PAAEKKK-EEXESKKEDSESDDEVMGFGLFD 127
Pglac-RPLP2-332 -----------------PGAADVEKILGSVGIEAXSEILKKVISXLNGKS-EELIAQGREKLSTMPVGGAAP--VAAVA---AAAA----------PAAE-KK-EDXESKKEESESDDEVMGFGLFD 127
Pglac-RPLP2-373 --------------------ADVEKILSSVGIEANFEKLKKVILELNGKNVEELIAQGREKLSSMPVGGAAP--VAVVA---AAAA----------PAAEKKK-EEXESKKEESESDDEVMGFGLLD 127
Pglac-RPLP2-226 -----------------PGADDVEKILSSVSVEANSEKLKKVISELNGKS-EELIAQGREKLSTMLVGGAAP--VAAVA---AAAA----------PAAEK-K-EEKETXKEES--DDEDMVFGLFD 127
Pglac-RPLP2-55  ------------------------KILNSVGVEANSEKLKKVISELNGKS-EELIAQGREKLSTMPVGGAAP--VAAVA---AAAA----------PVAE-KK-EEXESKKEESESDDEVMGFGLFD 127
Pglac-RPLP2-407 -----------------PGAADVEKILSSVGIEADSEKLKKVISXLNGKS-EELIAQGREKLSTMPVGGAAP--VAAVA---AAAA----------PAAEEKK-EEKEAKKEESXSDDEDMGFGLFE 127
Pglac-RPLP2-133 -----------------PGAADVEKILSSVGIEADSEKLKKVISELNGKS-EELIAQGREKLSTMPVGGAAP--VAAVA---AAAV----------PAAEEKK-EEKETKKEESXSDDEDMGFGLFD 127
Pglac-RPLP2-418 --------LAVLGGKASKGAADVVKILGSVGIEALNPRYXKVISELNGKS-EELIAQGREKLSTMPVGGAAP--VAAVA---ATAA----------PAAEKKK-EEXESKKEESESDDEVMGFGLFD 127
Pglac-RPLP2-67  -----------------PGAADVEKILSSMGIEAXSEILKKVISQLNGKS-EELIAQGREKLSTMLVGGAAP--VAAVA---AAAA----------PTAE-KK-EEXESKKEESESDDEVMGFGLFD 127
Pglac-RPLP2-37  -----------------PGAADVEKILSSVGIEANSEKLKKVISELNGKNVEELIAQGREKLSSMPVGGAAP--VAAVA---AAAA----------PAAEEKK-EEKETKKEESESDDEDMGFGLFD 127
Pglac-RPLP2-110 ------------------------KILNSVGVEANSEKLKKVISELNGKS-EELIAQGREKLSTMPVGGAAP--VAAVA---AAAA----------SPLRRRK---RESKKEESESDDEVMGFGLFD 127
Pglac-RPLP2-391 --------------------ADVEKILSSVGIEAXSEILKKVISELNGKS-EELIAQGREKLSTMPVGGAAP--VAAVA---AAAA----------PAAE-KK-EEXESKKEESESDDEVMGFGLFD 127
Pglac-RPLP2-107 -----------------PGAADVEKILSSVGIEAXSEILKKVISELNGKS-EELIAQGREKLSTMPVGGAAP--VAAAT---AAAA----------PAAEEKK-EEKETKKEES------------- 127
Pglac-RPLP2-14  -----------------PGAADVEKILSSMGIEADSEKLKKVISELNDKSVEELIAQGREKLSTMPVGGAAP--VAAVA---AAAA----------PAAEEKK-EEKETKKEESEFDDEDMGFGLFD 127
Pglac-RPLP2-314 ------------------------------GIEADSEKLKKVISELNGKSVEELIAXGREKLSTMPVGGAAP--VAAVA---AAAT----------PAAEEKN-DEXESKKEKSXSDDEDMGFGLFD 127
Pglac-RPLP2-4   ------------------------KILNSVGVEANSEKLKKVISELNGKS-EELIAQGREKLSTMSVGGAAP--VAAVA---AAAA----------PVAEKKK-EEXESKKEESESDDEVMGFGLFD 127
Pglac-RPLP2-167 ------------------------KILNSVGVEANSEKLKKVISELNGKS-EELIAQGREKLSTMPVGGTAP--VAAVA---AAAA----------PAAE-KK-EEXESKKEESESDDEVMGFGLFD 127
Pglac-RPLP2-337 --------LAVLGGKAFQGAADVEKITSSVGIEAXSEILKKVISELNGKS-EELTVQGREKLSTMPVGGAAP--VAAVA---AAAA----------PAAE-KK-EEXESKKQESESDDEVMGFGLFD 127
Pglac-RPLP2-276 -----------------PGAADVEKILSSVGIEANSEKLKKVISELNGKSVEELIAQGREKLSTMPVGGAAP--VAAVT---AAAA----------PAAEEKK-EEKETXKEES--DDEDMVFGLFD 127
Pglac-RPLP2-192 -----------------PGAADVEKILSSVGIEADSEKLKKVISELNGKS-EELIAQGREKLSTMPVGEAAP--VAAVA---AAAA----------PAAEEKK-EEKETKKEESQSDDEDMGFGLFD 127
Pglac-RPLP2-244 ------------------------KILNSVGVEANSEKLKKVISELNGKS-EELFAQGREKLSTMPVGGAAPVAVAAVA---AAAA----------PAAE-KK-EEXESKKEESESDDEVMGFGLFD 127
Pglac-RPLP2-105 ------------------GAADVEKILSSVGIEAXSEILKKVISELNGKS-EELIAQGREKLSTMPVGGAAP--VAAVA---AAAA----------PAAE-KK-EEXESKKEESESDDEVMGFGLFD 127
Pglac-RPLP2-277 -----------------PGAADVEKILSSVGIEADSEKLKKVISELNGKSVEELIAQGREKLSTMPVGGAAP--LVAVA---AAAA----------PAAEEKK-EEKETKKEESXSDDEDMGFGLFD 127
Pglac-RPLP2-125 -----------------PGAADVEKILSSVGIEADSKKLRKVISELNGRSVEELIAQGREKLSSMPVGGAAP--VAAVA---AAAA----------PAAEEKK-EEKETKKEESESDDEDMGFGLFD 127
Pglac-RPLP2-289 --------------------ADVEKILSSMGIEADSEKLKKVISEFNGKSVEELIAQGREKLSSMPVGGTAP--VATFA---AAAA----------PAAEEKK-EEKETKKEQSESDDEDMGFGLFD 127
Pglac-RPLP2-202 -------------------AADVEKILSSVGVEANSEKLKKVISELNGKSVEELIAQGREKLSTMPVGGAAP--VAAVT---AAAA----------PAAEE-K-EEKETKKEESSXDDEDMGFGLFD 127
Pglac-RPLP2-38  -----------------PGAADVEKILSSVGIEANSEKLKKVISELNGKS-EVLIAQGREKLSTMPVGGAAP--VAAVA---AAAA----------PAAEEKK-EEKETKKEEKETKKEE------- 127
Pglac-RPLP2-58  --------------------ADVEKILSSMGTEAXSEILKKVISELNGKS-EELIAQGREKLSTMPVSGAAP--VAAVA---AAAA----------PAAEKKK-EEXESKKEESESDDEVMGFGLFD 127
Pglac-RPLP2-176 -----------------PGAADVEKILSSVGIEANFEKLTKVISELNGKNVEELIAQGREKLSTMPVGGAAP--VAAVA---AAAA----------PAAEEKK-EEKETKKEESXSDYEDMGFGLFD 127
Pglac-RPLP2-283 -----------------PGAADVEKILSSVGIEANSEKLKKVISELNGKNVEELIAQGHAKLSSMPVGGAAP--VAAVA---AAAA----------PAAKEKK-EEKETKKEESXSDDEDMGFGLFN 127
Pglac-RPLP2-413 --------------------ADVEKILSSVGIEAXSEILKKVISELNGKS-EELIAQGREKLSTMPVGGTAP--VAAVA---AAAA----------PAAEKKK-EEXESKKEESESDDEDMGFGLFD 127
Pglac-RPLP2-215 ------------------------KILNSVGVEANSEKLKKVISELNGKS-EELIAQGREKLSTMPVGGAAPVAVAAVA---AAAA----------PAAE-KK-EEXESKKEESESDDEVMGFGLFD 127
Pglac-RPLP2-241 -----------------PGAADVEKILSSVGIEAXSEILKEVISELNGKS-EELIAQGREKLSTMPVGGAAP--VSAVA---AAAA----------PAAEKKK-EEXESKKEESESDDEDMGFGLFD 127
Pglac-RPLP2-171 ------------------------KILNSVDVEANSEKLKKVISELNGKS-EELIAQGREKLSTMSVGVAAP--VAAVA---AATA----------PAAE-KK-EEXESKKEESESDDEVMGFGLFD 127
Pglac-RPLP2-70  -----------------PGAADVEKILSSVGIEADSEXLKKVISELNSKS-EELIAQGREKLSTMPVGGVAP--VAVVA---AAAA----------SAAEEKK-EEKETXKDES--DDEDMVFGLFD 127
Pglac-RPLP2-205 -----------------PGAADVEKILSSVGVEANSEKLKKVISELNGKSVEELIAQGREKLSTMPVGGAAP--VAAVT---AAAA----------PAAEEKK-KEKETKKEESXSDDEDMGFGLFD 127
Pglac-RPLP2-345 --------------------AVVEKILRSVGIEADSKKLKKVVWELNGKSVEELIAQGRTKLSTMPVGGAAP--VAAVA---AAAA----------PAAEEKK-EEXESKKEESESDDEVMGFSLFD 127
Pglac-RPLP2-47  ------------------------KILNSVGVEANSEKLKKFISELNGKS-EELIAQGREKLSTMSVGGAAP--VAAVA---AAAA----------PVAEKKK-EEXESKKEESESDDEVMGFGLFD 127
Pglac-RPLP2-364 -----------------PGAAAVEKILSSVGIEADSEKLKKVISELNGKS-EELIAQGSEKLSSMPDGGVTP--VVAVA---AAAA----------LAAEEKK-EEKETKKEESXSDDEDMGLGLFD 127
Pglac-RPLP2-183 --------------------ADVEKILSSVGIEAXSEILKKVISELNGKS-EELIAQGREKLSTMPVGGAAP--VAAVA---AAAA----------PTAE-KK-EEXESKKEESESDDEVMGFGLFD 127
Pglac-RPLP2-309 -----------------PGAADVEKILSSVGIEADSEKLKKVISELNGNR-EELIAQGREKLSTMPVGGAVP--EAAVT---ATAA----------PAAEEKK-EEKETXKEESXSDDEDMVFGLFD 127
Pglac-RPLP2-88  -------------------AADVEKILSSVGIEADSEKLKKVISELNGKSVEELIAQGREKLSTMPVGGAAP--VAVVT---AAAA----------PAAEEKK-EEKETXKEES--DDEDMVFGLFD 127
Pglac-RPLP2-30  ------------------------KILNSVGVEANSEKLKKVISELNGKS-EELIAQGREKLSTMSVGGAAP--VAAVA---AAAA----------PVAE-KK-EEXESKKEESESDDEVMGFGLFD 127
Pglac-RPLP2-300 -----------------PGAADVEKILSSVGVEANSEKL-KVISELNGKSVEELIAQGREKLSTMPVGGAAP--MAAVA---AAAA----------PAAEEKK-EEKETKKEESXSDDEDMGFGLFD 127
Pglac-RPLP2-245 --------------------ADVEKILSSVGTEAXSEILKKVISELNGKS-EVLIAQGREKLSTMPVGGAAP--VAAVA---AAAA----------QAAE-KK-EEXESKKEESESDDEVMGFGLFD 127
Pglac-RPLP2-84  -----------------PGAADVEKILSSVGIEAXSEILKKVISELNGKS-EELIAQGREKLSTMPVGGAAP--VAAVA---AAAA----------PVAEKKK-EEXESKKEESESDDEVMGFGLFD 127
Pglac-RPLP2-135 -----------------PGAADVEKILSSVGIEAXSEILKKVISELNGKS-EELIAQVREKLSTMPVGXAAP--VAAVA---AAAA----------PAAEKKK-EEXESKKEESESDDEVMGFGLFD 127
Pglac-RPLP2-299 -----------------PGAADVEKILGSVGIEAXSEILKKVISXLNGKS-EDLIAQGREKLSTMPVGGAAP--VAAVA---AAAA----------PAAE-KK-EEXESKKEESESDDEVMGFGLFD 127
Pglac-RPLP2-431 --------LAVLGGKASQGAADVEKILSSVDIEAXSEILKKVISELNGKS-EELIAQGCEKLSTMPVGGAAP--VAAVA---AAAA----------PAAE-KK-EEXESKKEESESDDEVMGFGLFD 127
Pglac-RPLP2-148 ------------------------KILNSVGVEANSEKLKKVISELNGKS-EELIAQGREKLSTMPVGGAAP--VAVVA---AAAA----------PAAE-KK-EEXESKKEESESDDEVMGFGLFD 127
Pglac-RPLP2-284 -----------------PGAADVEKILSSLGIEANFEKLKKVISELNGENVEELIAQGREKLSSMPVGGAAP--VAAVA---AAAA----------PTGEKKK-EEXESKKEESESDDEVMGFGLFD 127
Pglac-RPLP2-392 -----------------PGAADVEKILSSVGIEAXSEILKKVISELNGKS-EELIAQGREKLLTMPVGGAAP--VAAVA---AAAA----------PAAEKKK-EEXESKKEESESDDEVMGFGLFD 127
                1.......10........20........30........40........50........60........70........80........90.......100.......110.......120.......
```

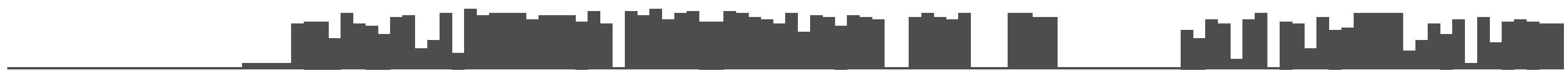

```
Pglac-RPLP2-294 -----------------PGAADVEKILSSVGIEANFEKLKKVISELNGKNVEELIAQGREKLSSMPVGGAAL--VAAVA---AAAA----------PAA-EKK-EEXESKKEESESDDEVMGFGLFD  127
Pglac-RPLP2-156 ------------------------KILNSVGVEANSEKLKKVISELNGKS-EELIAQGREKLSTMPFGGAAP--VTAVA---AATA----------PXAEKKK-EEXESKKEESESDDEVMGFGLFD  127
Pglac-RPLP2-207 -----------------PGAADVEKILSSVGVEANSEKLKKVISELNGKSVEELIAQGREKLSTMPVGGAAP--VAAVA---AAAA----------LAAEKKK-EEXESKKEESESDDEVMGFGLFD  127
Pglac-RPLP2-272 --------------------ADVEKILSSVGIEXESEILKKVISELNGKS-EELIAQGREKLSTMPVGGAAP--VATVA---AAAA----------PAAE-KK-EEXESKKEESESDDKVIGFGLFD  127
Pglac-RPLP2-62  -----------------PGAADVEKILSSVGIEADSEKLKKVISELNGKSVEELIAQGREKLSSMPIGGAAP--VAAIA---AAAA----------PAAEEKM---KDTKKEESESDDEDMGFGLFD  127
Pglac-RPLP2-356 -----------------PGTADVEKILSSVGIEADSEKLKKVILELNGKSVEELIAQGLEKLSSMPVGGAAP--VAAVA---AAAA----------PAAEEKK-EEKETXKEES--DDQDMVFGLFD  127
Pglac-RPLP2-36  --------LAVLGGKASQGAADVEKILSSVGIEAXSEILKKVISELNGKS-EELIAQGREKLSTMPVGGAAP--VAAVA---AAAA----------PAAE-KK-EEXESKKEESESDDEVMGFGLFD  127
Pglac-RPLP2-96  ------------------------KILNSVGVEANSEKLKKVISELNGNS-EELIAQGREKLSTMSVGGAAP--VAAVA---AAAA----------PVAEKKK-EEXESKKEESESDDEVMGFGLFD  127
Pglac-RPLP2-361 -----------------PSAADVEKIFSSVGIEANSEKLKKXILELNGKNVEELIAQGREKFSTMPVGGAAS--VAAVA---AAAA----------PAAEEKK-EEXETKKEESXSDDEDMGFGLFD  127
Pglac-RPLP2-158 -------------------------ILNSVGVEASSEKLKKVISEL--KS-EELIAQGREKLSTMPVGGAAP--VAAVA---AATA----------PXAEKKK-EEXESKKEESESDDEVMGFGLFD  127
Pglac-RPLP2-251 --------------------ADVEKILSSVGIEAXSEILKKVISELNGKS-EELIAQGREKLSTMPVGGVAP--VAAVA---AAAA----------PAAEKKK-EEXESKKEESESDVEVMGF----  127
Pglac-RPLP2-17  ------------------------KILNSVGVEANSEKLKKVISELNGKS-EELIAQGREKLSTMSVGGAAP--VAAVA---AAAA----------PVAEKKK-EEXESKKEESESDDEVMGFGLFD  127
Pglac-RPLP2-427 -----------------PGAADVEKILSSVGIEANFEKLKKVISELNGKNIEELIGQGREKLSSMPVGGAAP--VAAVA---AAAA----------PAAEEKK-EEKETKKEESXSDDEDMGFGLFD  127
Pglac-RPLP2-281 --------------------ADVEKILSSVGIEAXSEILKKVISELNGKS-EELIAQGREKLSTMPVGGAAP--VAAVA---AAAA----------PAAEKKK-EEXESKKEESXSDDEVMGFGLFD  127
Pglac-RPLP2-370 ------------------------KILNSVGVEANSEKLKKVISELNAKS-EKLIAQGREKLSTMPVGEQRP--WPWPR---SPLL----------QRRQLRR-RKRESKKEESESDDEFMGFGLFD  127
Pglac-RPLP2-198 ------------------------KILNSVGVEANSEKLKKVISELNGKS-EELIAQGREKLSTMPVGEAAP--VAAVA---AAAA----------PAAE-KK-EXRESKKKESESDDEVMGFGLFD  127
Pglac-RPLP2-293 -----------------PGAAGVEKILSSVGIEADSEKLKKVISELNGKSVEELIAQGREKLSSMPVGGAAP--MAAVA---AAAA----------PAAEEKK-EEKETKKEESESDDEDMGFGLFD  127
Pglac-RPLP2-121 ------------------------KILNSVGVEANSEKLKKVISELNGKS-EELIAQGREKLSTMPVGGAAP--VAAVA---AAAA----------PAAE-KK-EEXESKKEESESDDEVMGFGLFN  127
Pglac-RPLP2-71  -----------------PGAADVEKILSSVGIEADSEKLKKVISELNGKS-EELIAQGREKLSTMPVGGAAP--VAAVA---AAAA----------PAAEEKK-EEKETKKKESXSDDEVMGFGLFD  127
Pglac-RPLP2-428 --------------------ADVEKILSSVGIEANFEKLRKVISELNGKNVEELIVQGREKFSSMPVGGAAP--VATVA---AAAA----------PAAEKKK-EEXESKKEESESDDEVMGFGLFD  127
Pglac-RPLP2-153 ------------------------KILNSVGVEANSEKLKKVILELNGKS-EDLIAQGREKLSTMSVGGAAP--VAAVA---AAAA----------PVAE-KK-EEXESKKEESESDDEVMGFGLFD  127
Pglac-RPLP2-296 ------------------------KFLNSVGVEANSEKLKKVISELNGKS-EELIAQGREKLSTMPVGGAAP--VAAVA---AAAT----------PAAE-KK-EEXESKKEESESNDEVMGFGLFD  127
Pglac-RPLP2-116 ------------------------KILNSVGVEANSEKLKKVISELNGKS-EELFAQGREKLSTMPVGGAAPVAVATVA---AATA----------PAAE-KK-EEXESKKEESESDDEVMGFGLFD  127
Pglac-RPLP2-118 --------------------ADVEKILSSVGIEAXSEILKKVISELNGKS-EELIAQGREKLSTMPVGGAAP--VAAVA---AAAA----------PAAEKKK-EEXESKKEESESDDEVMGFGLFD  127
Pglac-RPLP2-404 ------------------------------GILQASKKLRKVISELNGKS-EELIAQGREKLSTMPVGGAAP--VAAVA---AAAA----------LAAEKKK-EEXESKKEESESDDEDMGFGLFD  127
Pglac-RPLP2-79  --------------------ADVKKILSSVGIEAXSEILKKVISELNGKS-EELIAQGREKLSTMPVGGAAP--VAAVA---AAAA----------PAAE-KK-EEXESKKEESESDDEVMGFGLFD  127
Pglac-RPLP2-268 ------------------------KILNSVGVEANSEKLKKVISELNGKS-EELIAQGREKLSTMSVGGAAP--VAAVT---AAAA----------PVAEKKK-EEXESKKEESESDDEVMGFGLFD  127
Pglac-RPLP2-63  -----------------PGAADVEKILSSVGIEADSEKLKKVISELNGKSVEELIAQGREKLSSMPVGGAAP--VAAVA---AAAA----------PTA-EKK-EEKETKKEESESDDEDMGFGLFD  127
Pglac-RPLP2-279 --------------------ADVEKILSSVGIEAXSEILKKVISELNGKS-EELIAQGREKLSTMPVGGAAP--VAAVA---AAAA----------PAAE-KK-EEXESKKEESESDDEVMGFGLFD  127
Pglac-RPLP2-321 -----------------PGAADVEKILSSVGIEADSEKLKKVISELNGKS-EELIAQGREMLSTMPVGGAAPAPVAAVA---AAAA----------PAAEKKK-EEKETKKEESESDDEDMVFGLFD  127
Pglac-RPLP2-243 -----------------PGTADVEKILSSVGIEADSEKLKKVISEFNGQSIEELIAQGGEELSSMPVGGAAP--VAAVT---AAAA----------PAAEEKK-EEKETKKEESESNDEDMGFGLFD  127
Pglac-RPLP2-408 -----------------PGAADVEKILSSVGIEADSEKLKKVISELNGKS-EELIAQGREKLSTMPVGGAAH--VAAVA---AAAA----------PAAEKKK-EEKETKKEESXSDDVDMGFGLFD  127
Pglac-RPLP2-415 -----------------PGAADVEKILSSVGIEENFEKLKKVISELNGKNVEELIAQGREKLSSMPVGGAAP--VAAVA---AAAA----------PAAEEKK-EEKKTKKGESXSDDEDMGFGLFD  127
Pglac-RPLP2-255 ------------------------KILNSVGVEANSEKLKKVISELNGKS-EELIAQGREKLSTMPVGGAAP--VAAVA---AAAA----------PAAE-KK-EEXESKKEESESDDEVMGFGLFN  127
Pglac-RPLP2-350 -------------------AADVKKILSSVGIEADSKXLKKVISELNGKSVEELIAQGREKLSTMPVGGAAP--VAAVA---TAAA----------PAAEXKK-EEKETKKEESDCDDEDMGFGLFD  127
Pglac-RPLP2-61  ------------------------KILNSVGVEANSEKLKKVISELNGKS-EELIAQGPEKLSTMSVGGAAP--VAAVA---AAAA----------PVAEKKK-EEXESKKEESESDDEVMGFGLFD  127
Pglac-RPLP2-136 -----------------PGAADVEKILSSVGIEADSERLKKVTSELNGKSIEELIAQGREKSSFTPVGGAAP--VAAVA---AAAA----------PAAEEKK-EEKETKKEESESDDEDMGFGLFD  127
                1.......10........20........30........40........50........60........70........80........90.......100.......110.......120.......
```

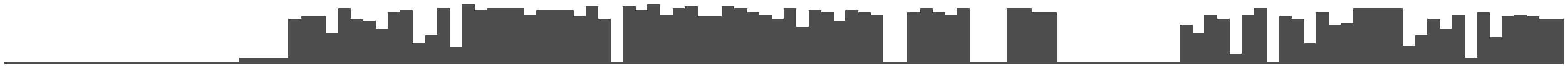

Supplement: Supplementary file 13 — Supplementary Data 10 [file 41467_2023_44023_MOESM13_ESM.pdf]
