# Supplementary material for: Dispersal from the Qinghai-Tibet plateau by a high-altitude butterfly is associated with rapid expansion and reorganization of its genome
Source: Nat Commun. 2023 Dec 11;14:8190. doi: 10.1038/s41467-023-44023-2 (PMC10713551; doi:10.1038/s41467-023-44023-2)
Supplement: Supplementary file 21 — Reporting Summary [file 41467_2023_44023_MOESM21_ESM.pdf]

nature portfolio

Corresponding author(s): NCOMMS-23-17976B

Last updated by author(s): Nov 1, 2023



# Reporting Summary

Nature Portfolio wishes to improve the reproducibility of the work that we publish. This form provides structure for consistency and transparency in reporting. For further information on Nature Portfolio policies, see our Editorial Policies and the Editorial Policy Checklist.

## Statistics

For all statistical analyses, confirm that the following items are present in the figure legend, table legend, main text, or Methods section.

| n/a | Confirmed | |
|---|---|---|
| ☐ | ☒ | The exact sample size ($n$) for each experimental group/condition, given as a discrete number and unit of measurement |
| ☐ | ☒ | A statement on whether measurements were taken from distinct samples or whether the same sample was measured repeatedly |
| ☐ | ☒ | The statistical test(s) used AND whether they are one- or two-sided<br>*Only common tests should be described solely by name; describe more complex techniques in the Methods section.* |
| ☐ | ☒ | A description of all covariates tested |
| ☐ | ☒ | A description of any assumptions or corrections, such as tests of normality and adjustment for multiple comparisons |
| ☐ | ☒ | A full description of the statistical parameters including central tendency (e.g. means) or other basic estimates (e.g. regression coefficient) AND variation (e.g. standard deviation) or associated estimates of uncertainty (e.g. confidence intervals) |
| ☐ | ☒ | For null hypothesis testing, the test statistic (e.g. $F$, $t$, $r$) with confidence intervals, effect sizes, degrees of freedom and $P$ value noted<br>*Give P values as exact values whenever suitable.* |
| ☐ | ☒ | For Bayesian analysis, information on the choice of priors and Markov chain Monte Carlo settings |
| ☐ | ☒ | For hierarchical and complex designs, identification of the appropriate level for tests and full reporting of outcomes |
| ☐ | ☒ | Estimates of effect sizes (e.g. Cohen's $d$, Pearson's $r$), indicating how they were calculated |

*Our web collection on statistics for biologists contains articles on many of the points above.*

## Software and code

Policy information about availability of computer code

| | |
|---|---|
| Data collection | SRA toolkit |
| Data analysis | 1. GCE v1.0.0<br>2. Hifiasm v0.13<br>3. BWA v0.7.12<br>4. ALLHiC<br>5. BUSCO v5.0.0<br>6. RepeatModeler v2.0.1<br>7. RepeatScout v1.0.5<br>8. RepeatMasker v4.0.6<br>9. Geneid v1.4<br>10. Genescan v1.0<br>11. GlimmerHMM v3.04<br>12. SNAP v2013<br>13. Augustus v2.4<br>14. Hisat v2.0.4<br>15. Stringtie v1.2.3<br>16. Transdecoder v2.0<br>17. Genemarks-t v5.1 |

18. JCVI 1.3.4
19. MAFFT v7
20. trimAl 1.4.1
21. RAxML v8.2.10
22. PAML v4
23. Orthofinder v2
24. Café v5
25. Figtree v1.4.4
26. Exonerate v2.2.0
27. Genewise v2.4.1
28. SAMtools v1.3.1
29. PSMC v0.6.5
30. GATK v4.0
31. Plink v1.9
32. Admixture v1.3.0
33. IQtree v2
34. Treemix v1.13
35. popgenWindows (https://github.com/simonhmartin/genomics_general/)
36. Jellyfish 2.2.10
37. edgeR 3.32.1
38. LDhelmet 1.10
39. selscan 2.0.0
40. BEAST v1.83
41. KaKs_Calculator v2.0
42. Vcftools v0.1.17
43. Bedtools v2.26.0

The script used in this work is available at Github (https://github.com/bioala/Parnassius-glacialis-genome-analysis).

For manuscripts utilizing custom algorithms or software that are central to the research but not yet described in published literature, software must be made available to editors and reviewers. We strongly encourage code deposition in a community repository (e.g. GitHub). See the Nature Portfolio guidelines for submitting code & software for further information.

## Data

Policy information about availability of data

All manuscripts must include a data availability statement. This statement should provide the following information, where applicable:

- Accession codes, unique identifiers, or web links for publicly available datasets
- A description of any restrictions on data availability
- For clinical datasets or third party data, please ensure that the statement adheres to our policy

The genome sequencing and assembled data have been deposited in the BioProject (PRJNA893814) of GenBank database. The Supplementary Figures and Tables in this study are provided in the PDF file of Supplementary Information. The Supplementary files in this study are provided in the files of Supplementary data 1-15. The Source data is provided as a Source Data file. The script used in this work is available at Github (https://github.com/bioala/Parnassius-glacialis-genome-analysis).

## Research involving human participants, their data, or biological material

Policy information about studies with human participants or human data. See also policy information about sex, gender (identity/presentation), and sexual orientation and race, ethnicity and racism.

| | |
|---|---|
| Reporting on sex and gender | Not Apply |
| Reporting on race, ethnicity, or other socially relevant groupings | Not Apply |
| Population characteristics | Not Apply |
| Recruitment | Not Apply |
| Ethics oversight | Not Apply |

Note that full information on the approval of the study protocol must also be provided in the manuscript.

# Field-specific reporting

Please select the one below that is the best fit for your research. If you are not sure, read the appropriate sections before making your selection.

☐ Life sciences ☐ Behavioural & social sciences ☒ Ecological, evolutionary & environmental sciences

# Ecological, evolutionary & environmental sciences study design

All studies must disclose on these points even when the disclosure is negative.

| | |
|---|---|
| Study description | Dispersal from the Qinghai-Tibet Plateau by a high-altitude butterfly is associated with rapid expansion and reorganization of its genome |
| Research sample | 1. Two 5th instar larvae of Parnassius glacialis;<br>2. 41 adult samples from 9 populations of Parnassius glacialis. |
| Sampling strategy | 1. For denovo genome sequecing: Two 5th instar larvae of Parnassius glacialis at the altitude range of 300m;<br>2. For genome re-sequecing: 9 populations of Parnassius glacialis at the altitude range of 300–1800 m. |
| Data collection | Genome and population sequencing. |
| Timing and spatial scale | Collect samples from western China to central and eastern China in 2021. |
| Data exclusions | No data were excluded in this study. |
| Reproducibility | The experimental findings can be repeated. |
| Randomization | The samples were collected randomly from the populations. |
| Blinding | Blinding was not relevant to our study.  These genome sequences from multiple populations objectively reflect the evolution information of Parnassius butterflies. |

Did the study involve field work? ☒ Yes ☐ No

## Field work, collection and transport

| | |
|---|---|
| Field conditions | Collect samples under the natural conditions in day-time. |
| Location | Information for the location of samples has been provided in manuscript and/or the additional files when necessary. |
| Access & import/export | The newly sequenced samples are widely distributed in China. This research complies with the commonly-accepted '3Rs' and the object herein is non-protective butterfly species, a lower invertebrate group. All samples were collected in the field. No permits were required for collection of these species. |
| Disturbance | No disturbance caused by this study. |

# Reporting for specific materials, systems and methods

We require information from authors about some types of materials, experimental systems and methods used in many studies. Here, indicate whether each material, system or method listed is relevant to your study. If you are not sure if a list item applies to your research, read the appropriate section before selecting a response.

## Materials & experimental systems

| n/a | Involved in the study | |
|---|---|---|
| ☒ | ☐ | Antibodies |
| ☒ | ☐ | Eukaryotic cell lines |
| ☒ | ☐ | Palaeontology and archaeology |
| ☐ | ☒ | Animals and other organisms |
| ☒ | ☐ | Clinical data |
| ☒ | ☐ | Dual use research of concern |
| ☒ | ☐ | Plants |

## Methods

| n/a | Involved in the study | |
|---|---|---|
| ☒ | ☐ | ChIP-seq |
| ☒ | ☐ | Flow cytometry |
| ☒ | ☐ | MRI-based neuroimaging |

## Animals and other research organisms

Policy information about studies involving animals; ARRIVE guidelines recommended for reporting animal research, and Sex and Gender in Research

| | |
|---|---|
| Laboratory animals | Not Apply |
| Wild animals | 5th instar larvae and adult samples of Parnassius glacialis. |
| Reporting on sex | NA |
| Field-collected samples | For laboratory work with field-collected samples, all relavant parameters have been provided in the part of Materials and methods of manuscript. |
| Ethics oversight | No permits were required for collection of these species. |

Note that full information on the approval of the study protocol must also be provided in the manuscript.
